# A low cost, low power sap flux device for distributed and intensive monitoring of tree transpiration

**DOI:** 10.1016/j.ohx.2022.e00351

**Published:** 2022-08-27

**Authors:** Justin Beslity, Stephen B. Shaw, John E. Drake, Jason Fridley, John C. Stella, Jordan Stark, Kanishka Singh

**Affiliations:** a SUNY College of Environmental Science & Forestry, Syracuse, NY, USA; b Syracuse University Syracuse, NY, USA; c Cornell University, Ithaca, NY, USA

**Keywords:** Sap flux, Heat pulse velocity (HP), Low-cost sensors

## Abstract

Accurate estimation of transpiration in individual trees is important for understanding plant responses to environmental drivers, closing the water balance in forest stands and catchments, and calibrating earth system models, among other applications. However, the cost and power consumption of commercial systems based on sap flow methods still limit their usage. We developed and tested a cost-effective (<$150), simple to construct, and energy efficient sap flux device based on the heat pulse method. Energy savings were achieved by reducing the voltage of heat pulses and using an internal clock to completely shut down the device between pulses. Device accuracy was confirmed by laboratory estimates of sap flow made on excised branches of *Acer saccharum* and *Tsuga canadensis* (adjusted R^2^ = 0.96). In a 174-d field installation of 12 devices, batteries (eight rechargeable Ni-MH AA) needed to be replaced every 14 days. Sap flux measurements in the field tracked expected variations in vapor pressure deficit and tree phenology. The low cost, compact design, reliability, and power consumption of this device enable sap flux studies to operate with more replication and in more diverse ecological settings than has been practical in the past.

## Graphical abstract

**Figure:**
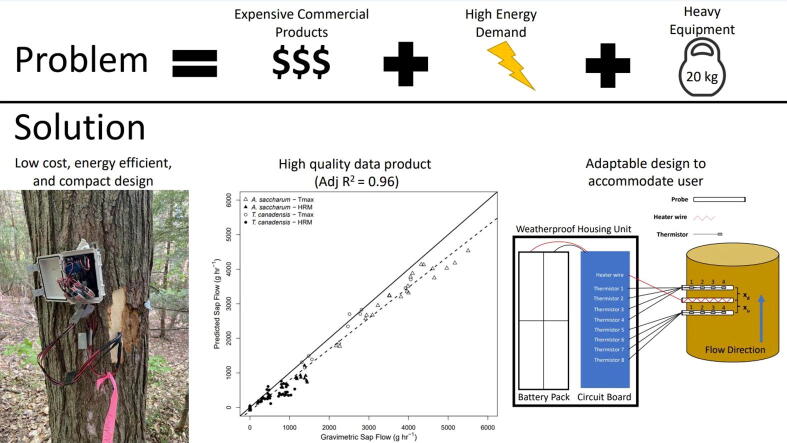

**Specifications table:** 

Hardware name	J_s_^5^
Subject area	Environmental, Planetary and Agricultural Sciences
Hardware type	• Field measurements and sensors • Electrical engineering and computer science
Open-Source License	GNU General Public License v3.0
Cost of Hardware	Complete unit: $109
Source File Repository	OSF: https://doi.org/10.17605/OSF.IO/KBZ5Y GitHub: https://github.com/justinbeslity/Js5-Novel_Sap_Flux_Device

## Hardware in context

Canopy transpiration (E_c_) from trees is the largest component of evapotranspiration (ET) in many habitats [1], [2], [3], and the measurement of E_C_ is critical to understanding vegetative, hydrologic, and meteorologic function in forested systems. Several methods have been developed to measure E_c_, including sap flow measurements, weighing lysimeter, and eddy covariance [1], [4], [5], [6]. Despite the wide application of these methods, they remain expensive, time consuming, and cumbersome to employ [7], [8]. Advancement of sap flow methods offer perhaps the most promising route for simplifying implementation and reducing costs of Ec measurements. However, since sap flow measurements occur at the scale of a single tree, attempts to estimate E_c_ over a large area must involve the measurement of sap flow from multiple trees and at different radial depths in order to properly capture variations in transpiration due to tree size, species, and landscape position [9], [10], [11], [12]. This poses a fundamental constraint for large scale transpiration scaling, as the required number of sap flow sensors is often prohibitively expensive if purchased from commercial retailers (i.e., ≥$1000 per unit). Cost-saving options such as using centralized data loggers within plots make it difficult to select trees that adequately represent the study watershed, while commercial probes do not allow for sampling a wide range of radial depths or with different sap flux methods. In addition to cost, energy consumption is a concern. Sap flow installations away from the convenience of the electrical grid have traditionally used heavy and bulky lead-acid batteries, either replaced every few days or connected to solar panels [13], [14], [15]. The need for frequent transport of bulky batteries places an additional logistical limit on the number of sensors that can be deployed and limits the ability to monitor for extended periods.

To overcome these two primary constraints of cost and energy usage, we developed and tested a prototype sap flow device, hereafter referred to as the J_s_^5^ (a play on the common abbreviation for sap flux density and the developers’ names), designed to be low cost (<$150), adaptable, and to draw substantially less power than standard, existing commercial devices, while maintaining a high degree of accuracy. Several recent attempts have been made to develop custom-built devices to circumvent the need for commercial probes and dataloggers. Cárdenas et at. (2019), Miner et al. (2017), and Jones et al. (2020) each developed sap flow units using cost-efficient, microcontroller-based designs [16], [17], [18]. However, none of these devices implemented measures to prolong battery life. Cárdenas et al. (2019) and Miner et al. (2017) reported that the devices could run for several days to a week using a 12V lead acid battery and a 14.8V lithium-ion battery respectively, which would limit field applications and require frequent battery changes. Jones et al. (2020) powered their device using a 12V lead acid battery, and their device has a nominal continuous power demand of 85mW. Additionally, none of these prior devices demonstrated an ability to work over the range of expecting field conditions in trees.

Here, we describe the development and evaluation of two features to reduce power consumption in sap flow sensors, including a) the minimum voltage of the applied heat pulse necessary to accurately measure sap flux density (SFD), and b) the ability to use an internal clock to allow for zero power consumption between measurements. In addition to the power savings offered by this device, we demonstrate its flexibility by sampling across several radial depths using two different sap flux methods. The overall effectiveness of the J_s_^5^ as well as the power savings and reliability are evaluated using species of different xylem anatomy (*Tsuga canadensis* [softwood], and *Acer saccharum* [diffuse-porous hardwood]) in both lab tests (using excised branches) and field tests.

## Hardware description

**Fig. 1:**
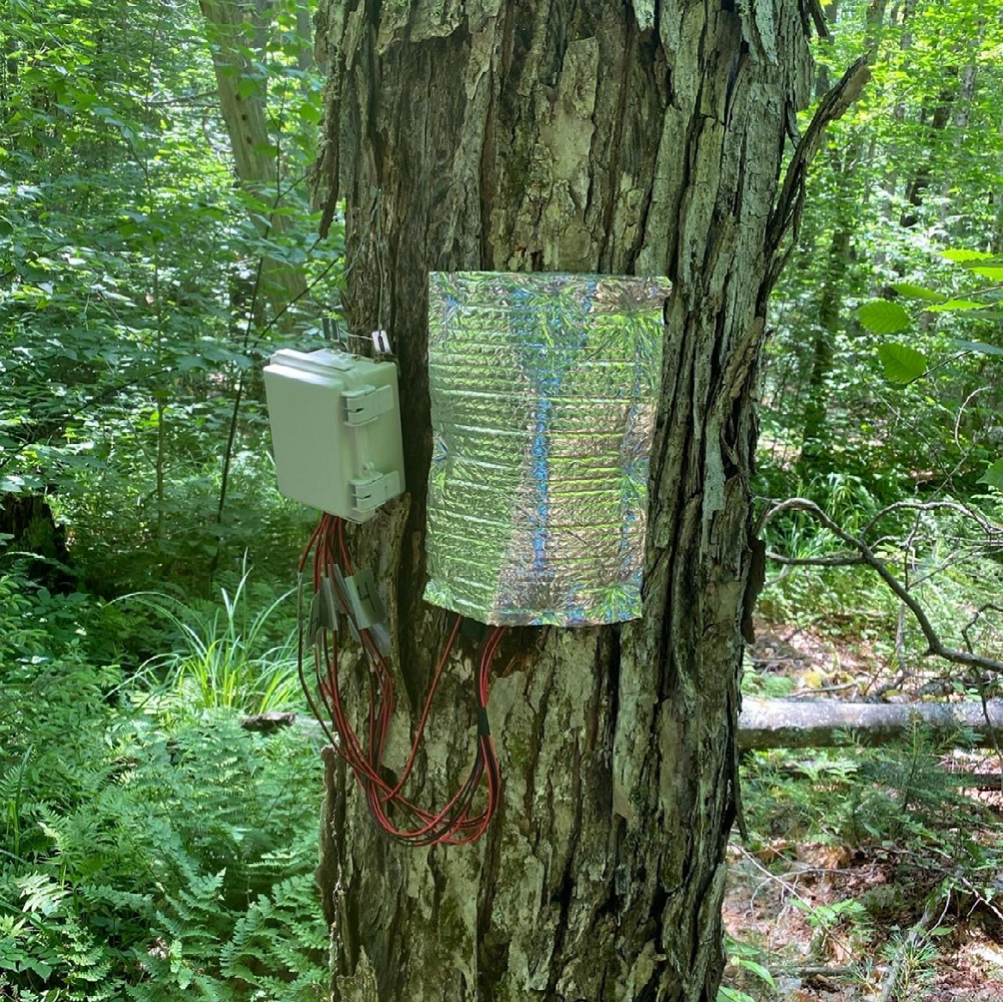
J_s_^5^ deployed in the field. The waterproof housing case contains both the printed circuit board, as well as the eight AA batteries used to power the device. The probes extend out of the bottom of the housing unit and are inserted into drilled holes in the tree. Mylar shielding is placed over the probes to reduce the influence of solar radiation. Photo credit**.** Justin Beslity.

The J_s_^5^ consists of two main components: 1) probes used for generating and detecting heat differences and 2) circuitry (Fig. 1).

### Probes

All probes were constructed with 5 cm long stainless-steel dispensing needles. Reference probes (i.e., temperature sensor probes) were constructed from 1.27-mm diameter (18 gauge) needles, and a 10*K* NTC thermistor was used for the temperature sensor (GA10K2MCD1, TE Connectivity, Schaffhausen, Switzerland). Multiple thermistors can be placed within a single dispensing needle to obtain multiple radial measurements across the same reference probe. Thermistor resistance was converted to temperature using the Steinhart-Hart equation. Heating probes were constructed by threading two loops of heater wire (36AWG [0.12-mm], TFCC Insulated Thermocouple Wire, Omega Engineering Inc., Norwalk, CT, USA) through the needle [16]. Approximately 25 cm of heater wire are required to construct a 5 cm long heater probe. All probes were backfilled with epoxy (Omega Bond 101, Omega Engineering, Stamford, CT, USA) once thermistors or heater wires were inserted.

### Circuitry

The controller and data logger design are adapted from Miner et al. (2017) [16]. An ESP8266-based microcontroller (Lolin D1 mini, WeMos Electronics) is used to operate the sap flow probes. This microcontroller offers a cost-effective replacement to both the Campbell datalogger and Arduino UNO in a compact size which could fit within a compact, custom, weather-proof housing unit (Polycase, Inc.). Data is recorded using a micro-SD card inserted into a card reader module connected to the microcontroller. A P-Channel MOSFET (IRF9540) and a real time clock (RTC; DS3231) act as a switch to turn off the microcontroller, allowing for complete system shutdown, a major power saving innovation [19]. The RTC maintains date-time information with a separate 3.3V coin battery (CR2023) during system shut down. The input voltage is moderated by the P-Channel MOSFET. The RTC square-wave output signal is used to close the P-Channel MOSFET gate and complete the circuit during each sampling cycle. A *N*-Channel MOSFET, controlled via the microcontroller, is used to operate the heater probes. Input voltage is moderated via DC linear fixed voltage regulators to provide a constant and rated output voltage. All components were mounted on a custom PCB and soldered in place. The entire system is powered by eight AA Ni-MH rechargeable batteries.

### Software system

The software system for the J_s_^5^ includes two separate programs, both coded with Arduino IDE [20]. The first program sets the date and time on the RTC unit, as well initiates the SD card, establishing the file to which the data will be written. The second program is designed to:

1. Initiate the microcontroller.
2. Collect baseline temperature measurements via the 10*K* NTC thermistors placed within the reference probes.
3. Initiate the heat pulse (∼ 2 s).
4. Monitor the subsequent temperature changes (∼ 100 s).
5. Clear the alarm on the RTC to open the P-Channel MOSFET gate and power off the device.

This sequence then repeats based on the two programable alarms set on the RTC. While a 30 min sampling rate is common for sap flow measurements, the RTC can be programmed to sample at more or less frequent rates [21], [22], [23].

## Design files

**Table:** 

**Design file name**	**File type**	**Open-source license**	**Location of the file**
Gerber_Sap-Flux-System_SD-Card_8-Therms-042221_2022-03–21	Gerber	GNU General Public License v3.0	https://doi.org/10.17605/OSF.IO/KBZ5Y
Board_Configuration_SD.ino	INO	GNU General Public License v3.0	https://doi.org/10.17605/OSF.IO/KBZ5Y
Write-Sap-Flow-Code_SD_Card.ino	INO	GNU General Public License v3.0	https://doi.org/10.17605/OSF.IO/KBZ5Y

1. Gerber_Sap-Flux-System_SD-Card_8-Therms-042221_2022-03-21: The file used to order designed printed circuit board via JCLPCB (PCB Prototyping and Manufacturing, Guangdong Province, China).
2. Board_Configuration_SD.ino: The file used to program the RTC unit and initialize the SD card.
3. Write-Sap-Flow-Code_SD_Card.ino: The file used to run sap flux measurment operations.

## Bill of materials

**Table:** 

**Designator**	**Part**	**Number**	**Cost per unit - USD**	**Total Cost - USD**	**Distributer**	**Material Type**
Thermistors	10*K* NTC Thermistor (GA10K3MCD1)	8	$2.89*	$23.12	Mouser	Thermistor
Microcontrollers	Lolin Wemos D1 Mini	1	$7.50	$7.50	Amazon	Non-specific
ADC	ADS1115	2	$6.00	$12.00	Amazon	Non-specific
RTC	DS3231SN	1	$3.60	$3.60	Amazon	Non-specific
2.5 V Reference	LT1460GIZ-2.5#PBF	1	$2.35*	$2.35	Mouser	Non-specific
6 V Regulator	L7806CV-DG	1	$0.31*	$0.31	Mouser	Non-specific
P-Channel MOSFET	IRF9540NPBF	1	$0.72*	$0.72	Mouser	Semi-conductor
*N*-Channel MOSFET	RFP30N06LE	1	$0.80*	$0.80	Amazon	Semi-conductor
MicroSD Card Adapter	MicroSD Card Adapter (HW-125)	1	$1.00	$1.00	Amazon	Non-specific
MicroSD Card	SanDisk Extreme 32GB microSD	1	$15.00	$15.00	Amazon	Non-specific
Battery Holders	8x AA Battery Holders	1	$8.18*	$8.18	Amazon	Non-specific
AA Batteries	Energizer Ni-MH 1.2 V 2.3 Ahr batteries	8	$1.88	$15.00	Amazon	Ni-MH
Lithium Coin Cell Batteries	Lithium CR2032 3 V batteries	1	$0.60	$0.60	Amazon	Lithium
Outer Protective Casing	WH-02 winged NEMA enclosure	1	$8.00	$8.00	Polycase	Non-specific
1K ohm Resistors	1K ohm resistors	1	$0.01	$0.01	Amazon	Resistor
10K ohm Resistors	10K ohm resistors	11	$0.11	$1.21	Amazon	Resistor
100k ohm Resistors	100K ohm resistors	1	$0.11	$0.11	Amazon	Resistor
Heater Wire	TFCC-005 PFA insulated wire	< 2 ft	$1.10	$1.10	Omega	Resistance Wire
Stainless Steel Dispensing Needle	75165A249	3	$0.56	$1.69	McMaster-Carr	Non-specific
Jumper Wires (red)	Hellotronics 15 cm M/M red	10	$0.12	$1.20	Amazon	Non-specific
Jumper Wires (black)	Hellotronics 15 cm M/M black	10	$0.12	$1.20	Amazon	Non-specific
Extension wire	24 AWG Low Voltage Red/Black bonded wire	∼ 5 ft	$2.00	$2.00	McMaster-Carr	Insulated copper wire
Electrical Tape	Scotch 3M Electrical Tape	Small Amount	$1.00	$1.00	Amazon	Non-specific
Epoxy	OB-101-2	Small Amount	$0.30	$0.30	Omega	Non-specific
Heat shrink wrap	PPCLION 3/8 and 1/8 in 3:1 heat shrink wrap	Small Amount	$1.00	$1.00	Amazon	Non-specific

Pricing Notes:

* price will vary with distributor and purchase date.

## Build instructions

**Fig. 2:**
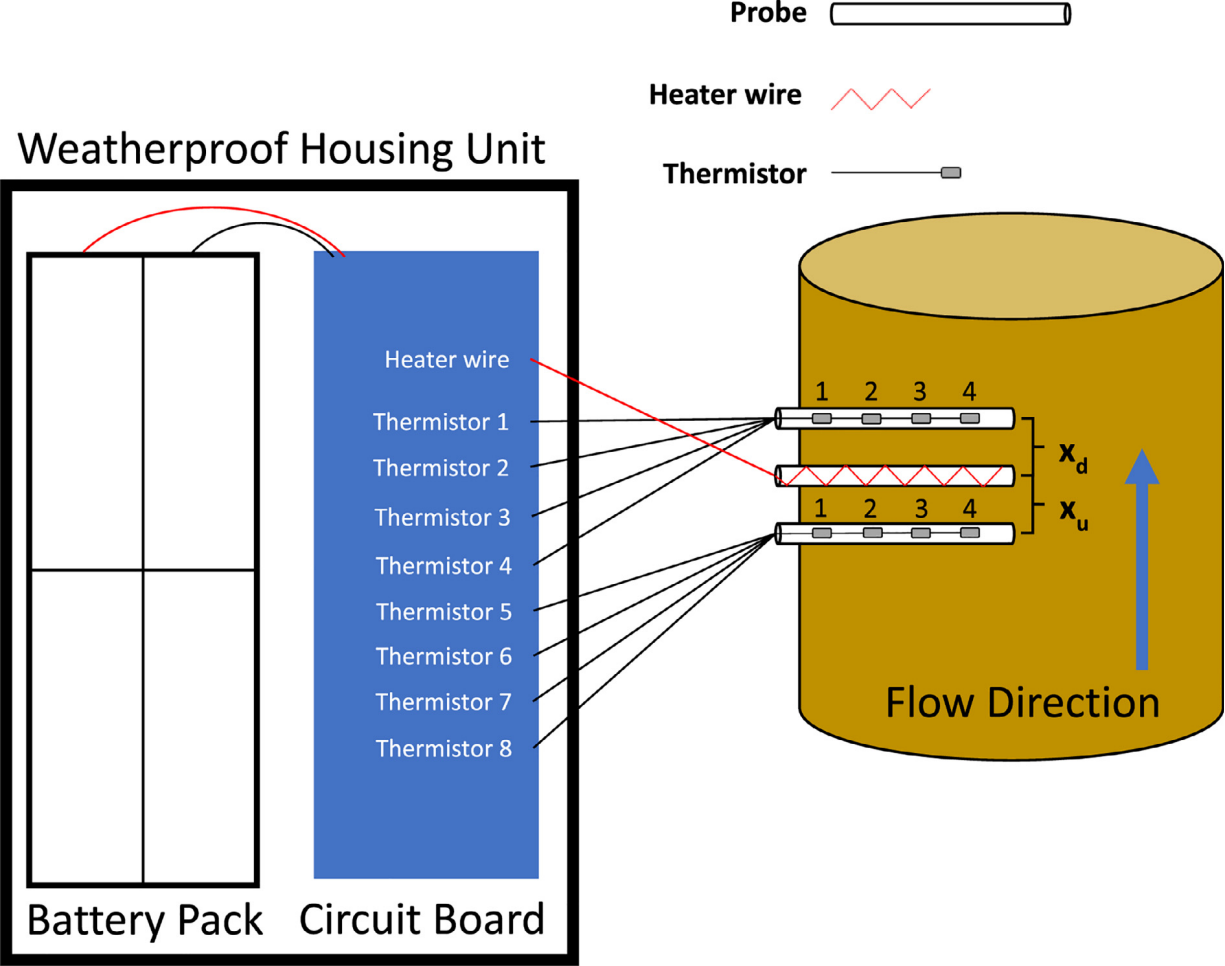
Simplified schematic representation of the J_s_^5^. The thermistor numbers on the circuit board correspond to the numbers above the thermistors in the reference probes (i.e., temperature sensor probes). The circuit board is designed to accommodate eight thermistors, which allows for four measurement depths in most sap flow methods. The spacing between heater probe and the downstream (x_d_) and upstream (x_u_) reference probes can be adjusted to switch between different sap flow methods. The 8x AA battery holder and printed circuit board fit side by side within the waterproof housing unit (WH-02 winged NEMA enclosure).

The hardware needed to construct the J_s_^5^ is listed in the Bill of Materials. The device can be separated into to two main components, 1) the probes, and 2) the printed circuit board (PCB) (Fig. 2). The probes and circuit board can be customized to meet the needs of a particular study (i.e., number of radial measurement points, length of the probe, sampling frequency, and sap flow methodology).

### Probes

Most commercial sensors include two radial measurement points along the length of the probe (e.g., Implexx Sap Flow Sensor, Implexx Sense, Melbourne, Victoria, Australia). At least two radial measurements are recommended when using the heat pulse velocity method, though specific experiments may require additional radial measurement points for more accurate measurements [24]. Here, the construction of a 5 cm reference probe with four radial measurement points (0.5, 1.5, 2.5, 3.5 cm) will be detailed, as well as the accompanying 5 cm heater probe.

**Fig. 3:**
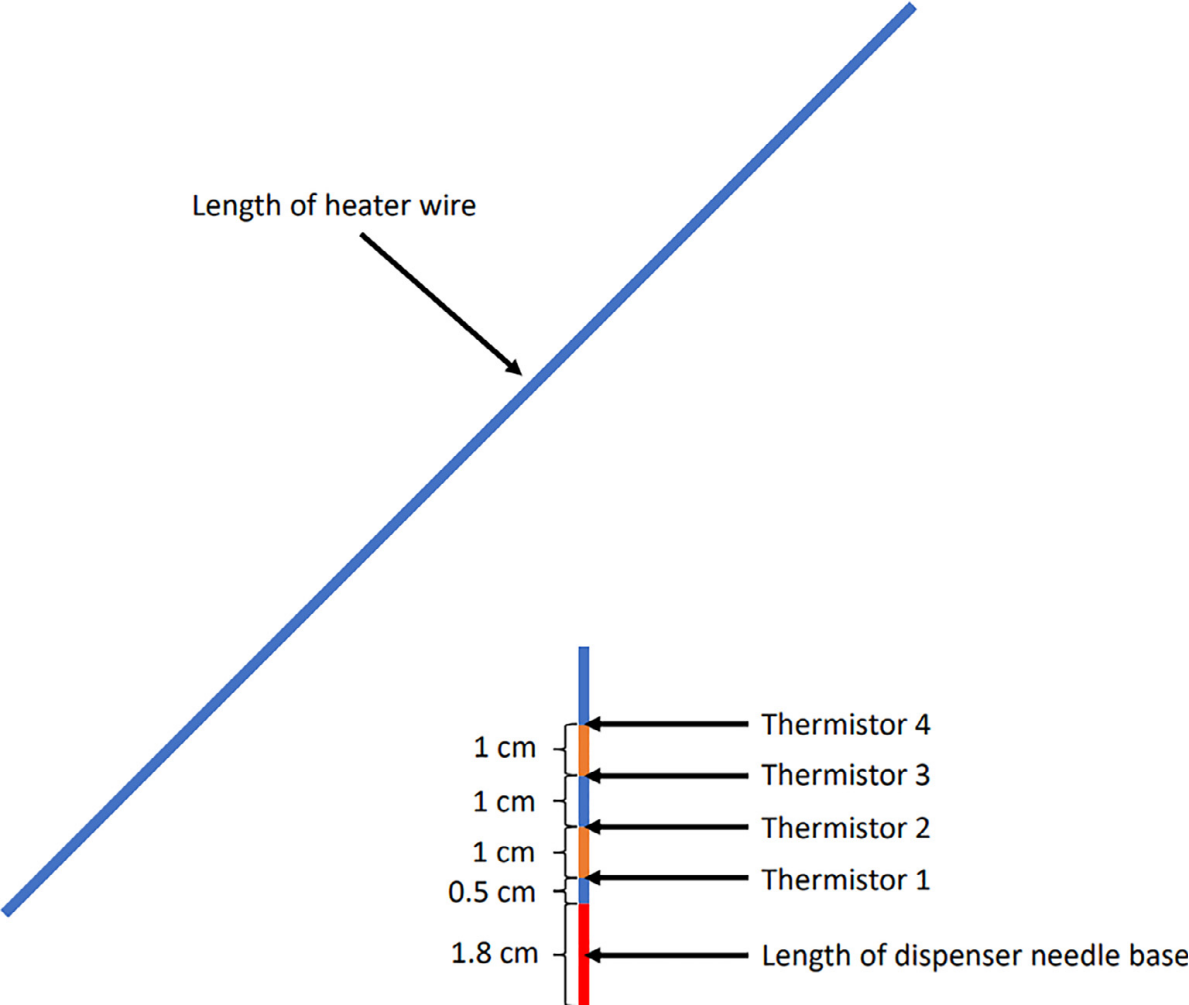
PowerPoint template of thermistor placement withing dispensing needle and the necessary length of heater wire needed for the corresponding heater probes. The placement of thermistors is dependent on the needs of the user and should be adjusted depending on the intended use. Typically, at least two thermistors at different radial depths are recommended. Figure not to scale.

**Fig. 4:**
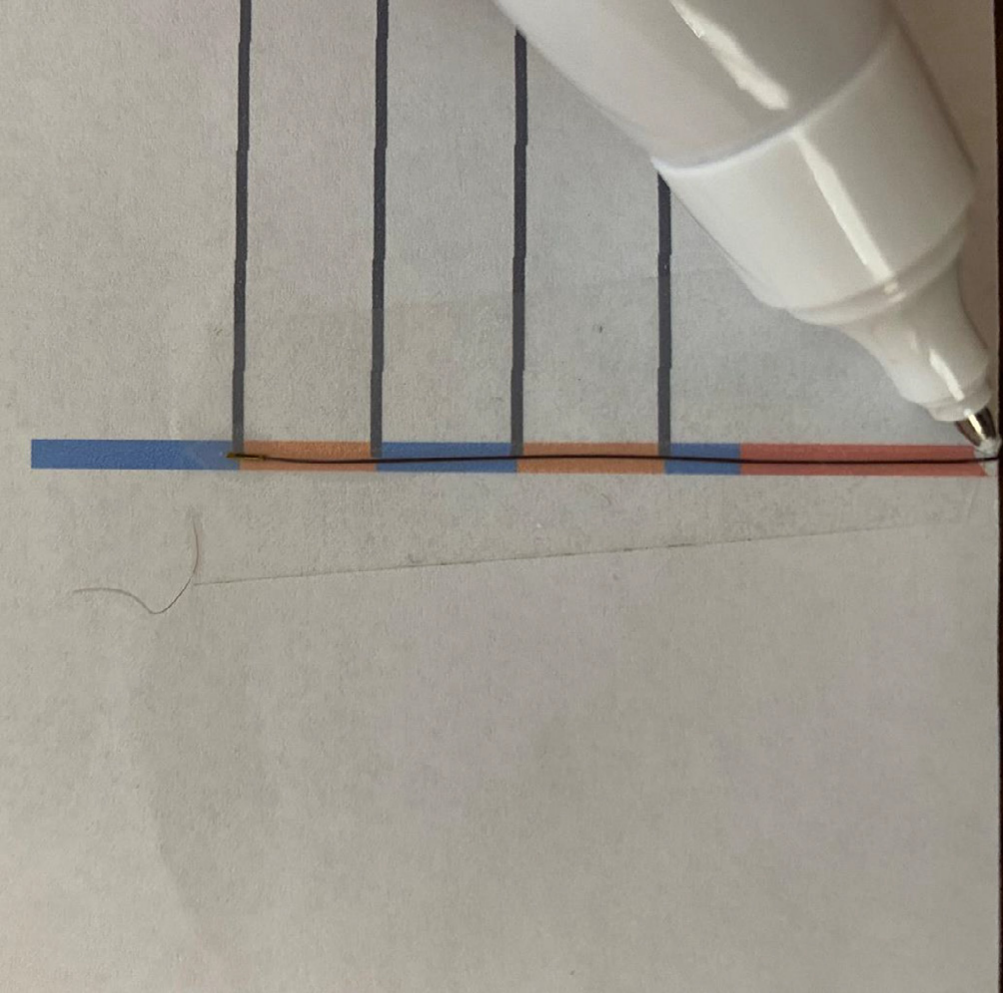
Marking the thermistor using white out pen. The thermistor bead has been positioned at the mark corresponding to “Thermistor 4” on Fig. 2. The thermistor was taped down for this picture, and this is not recommended during construction. Multiple thermistors can be marked concurrently to expedite this step.
  c. *Align thermistors in dispensing needle:* Starting with the thermistor located at the deepest point within the dispensing needle (in this example, Thermistor 4), insert the thermistors through the base of the dispensing needle and align the applied white out mark of each thermistor with the edge of the bottom of the base (Fig. 5). Once each thermistor is properly placed, apply a tiny bead of super glue to temporarily hold the thermistors lead wires in place. Allow time to dry.

1. Reference Probe (i.e., temperature probe)
  a. *Cut dispensing needle to desired length (optional):* When measuring sap flow on smaller diameter trees, it may be desirable to cut the dispensing needles to a shorter length. Using either a hacksaw or a Dremel (recommended), cut the shaft of the dispensing needles to the desired length. In this example, the entire 5 cm length of the dispensing needle is used, and no cutting is required.
  b. *Mark thermistor depth:* A 1:1 scale template, created with PowerPoint, depicting the location of each thermistor bead within the dispensing needle is recommended to accurately place thermistors (Fig. 3). Using the PowerPoint template, align the thermistor bead at the corresponding depth, making sure to account for the length of the dispensing needle base (1.8 cm), and mark the lead wires using a fine point white out pen (Fig. 4). Repeat this process for each thermistor at each depth.

**Fig. 5:**
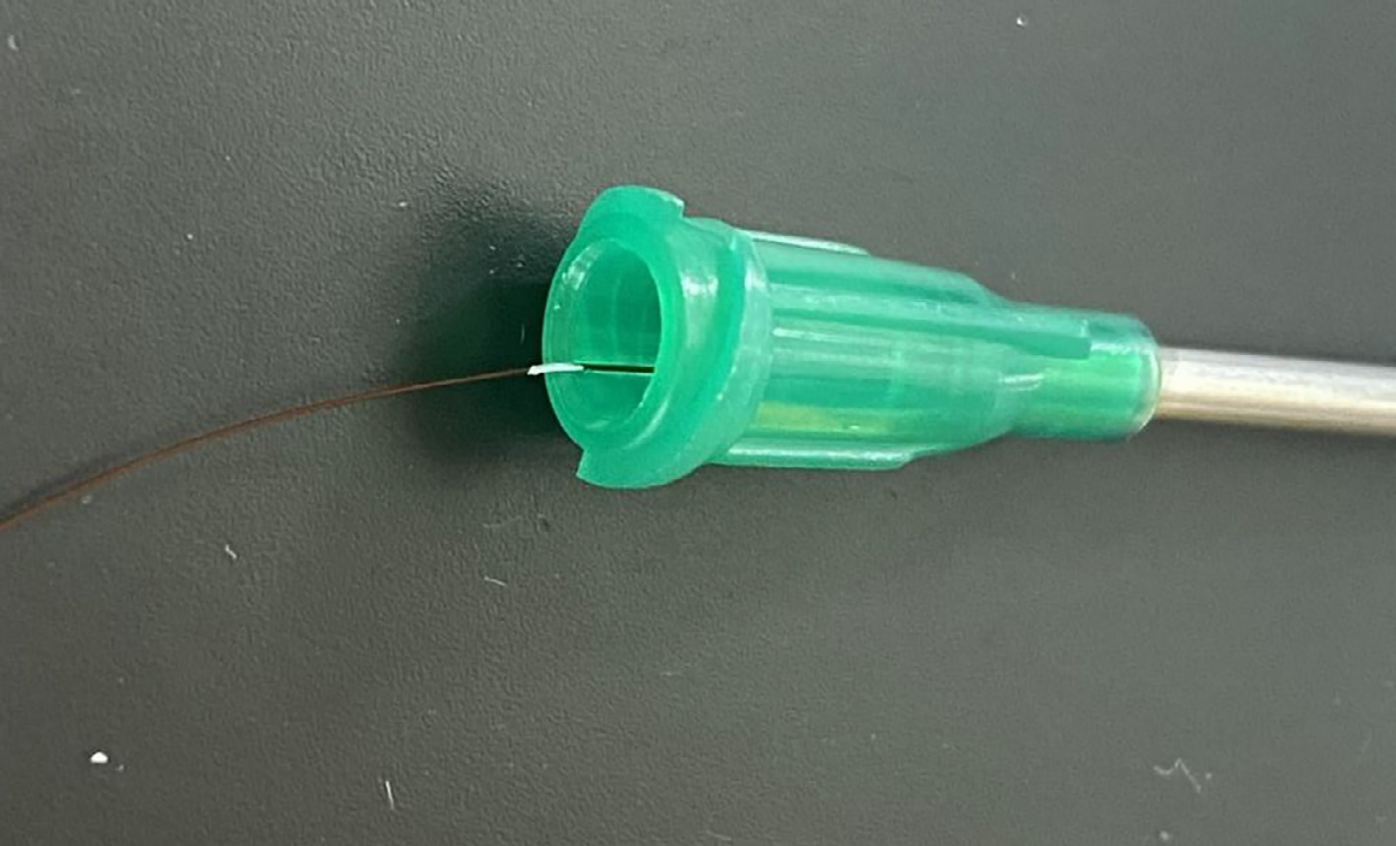
Aligned thermistor in dispensing needle. Note the applied white out mark aligned with the edge of the bottom of the dispensing needle base.
  d. *Prepare epoxy:* When ready, mix equal portions of the Omega Bond 101 resin and hardener in a weigh boat. Epoxy will become difficult to work with after one hour and completely sets in 24 h.
  e. *Dispense epoxy:* Load the 1 mL syringe with epoxy and firmly insert the tip of the syringe into the base of the prepared dispensing needles. The super glue will provide some security, but it may be helpful to bend the thermistor lead wires back upon the dispensing needle base and hold them in place (Fig. 6.a). Dispense epoxy until a small amount emerges from the tip of the dispensing needle. Remove the excess epoxy from the tip of the needle, ensuring that the length of the metal shaft is free from epoxy. Very gently, work the syringe tip out of the base of the dispensing needle. Fill in the base with additional epoxy (Fig. 6.b). Take care to keep the thermistor lead wires free of epoxy. Let cure for 24 h.

**Fig. 6:**
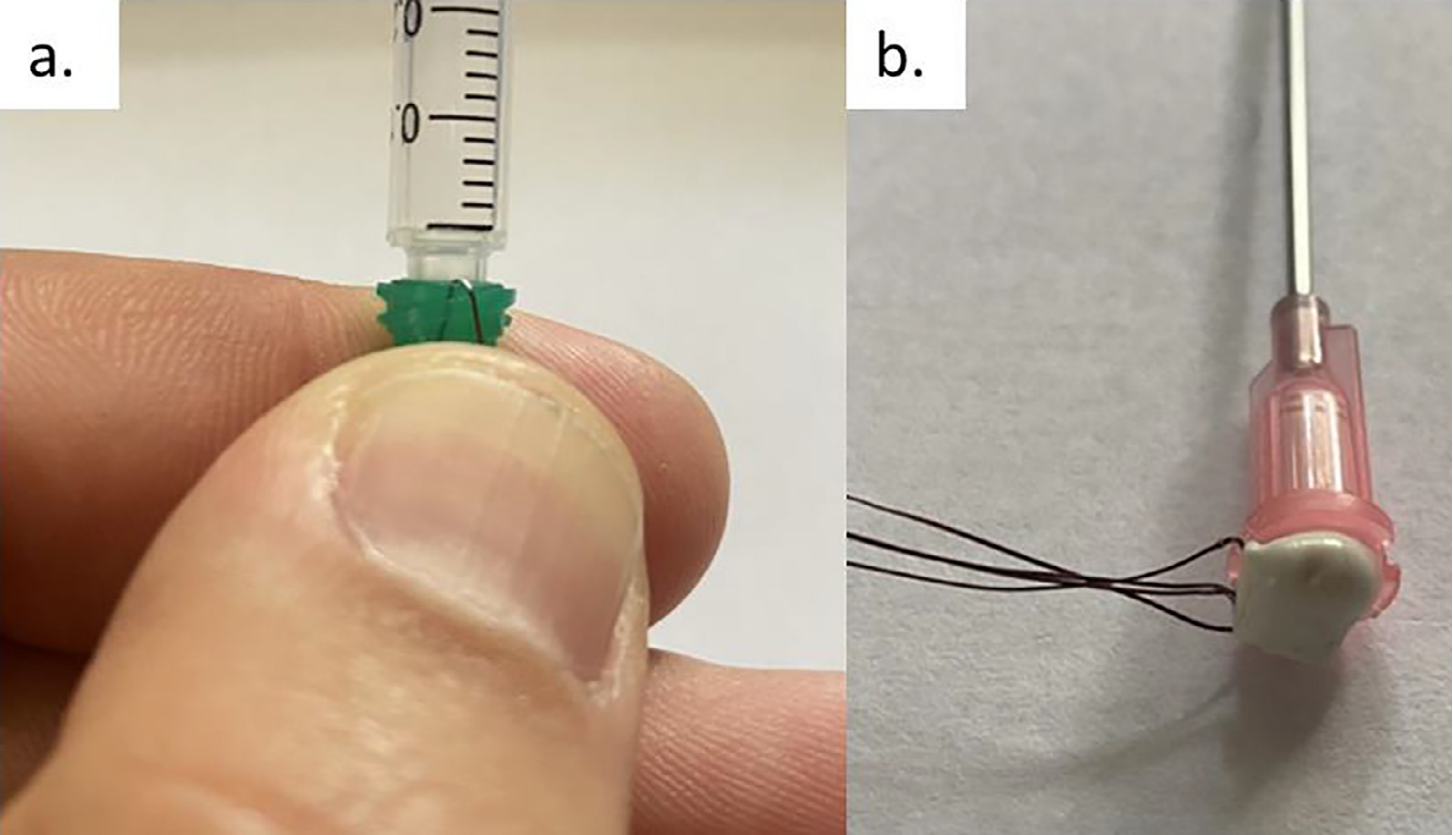
Applying epoxy through the base of the dispensing needle. While the small amount of super glue applied in section c. *Align thermistors in dispensing needle* will keep the thermistors secured to the base, it may be helpful to bend the thermistors back against the base of the dispensing needle when inserting the 1 mL syringe into the base of the syringe, as seen in Fig. 6.a. After observing the epoxy emerging from the tip of the needle, and while continuing to hold the thermistors in place, gently work the syringe tip out of the base of the dispensing needle and apply additional epoxy to fill the base of the dispensing needle, as seen in Fig. 6.b.
  f. *Attach extension wire to thermistor leads:* Once the epoxy has cured, separate the two lead wires of each thermistor (notice the small, uninsulated ends of each lead wire). Thermistors can be identified by the length of the lead wires protruding from the base of the dispensing needle (Fig. 7). Using a small piece of duct tape as a label, number four ∼ 60 cm segments of 24 AWG low voltage bonded extension wire from one to four corresponding with the thermistors in the dispensing needle (Fig. 8). Labeling the bonded extension wire corresponding to the connected thermistor is critical as there is no simple way to differentiate the thermistors once the probe has been completed. Strip the ends (∼ 0.5 cm) of each segment of bonded wire, place a 1 cm piece of the 1/8 in diameter shrink wrap on the bonded wire, firmly wrap the thermistor lead wire around the exposed bonded wire. Solder the exposed lead of the thermistor and the bonded extension wire together, move the small piece of heat shrink wrap over the exposed wire, and use a heat gun to seal the connection (Fig. 9). Alternatively, electrical tape can be used to insulate the connections. Repeat this process for each thermistor.

**Fig. 7:**
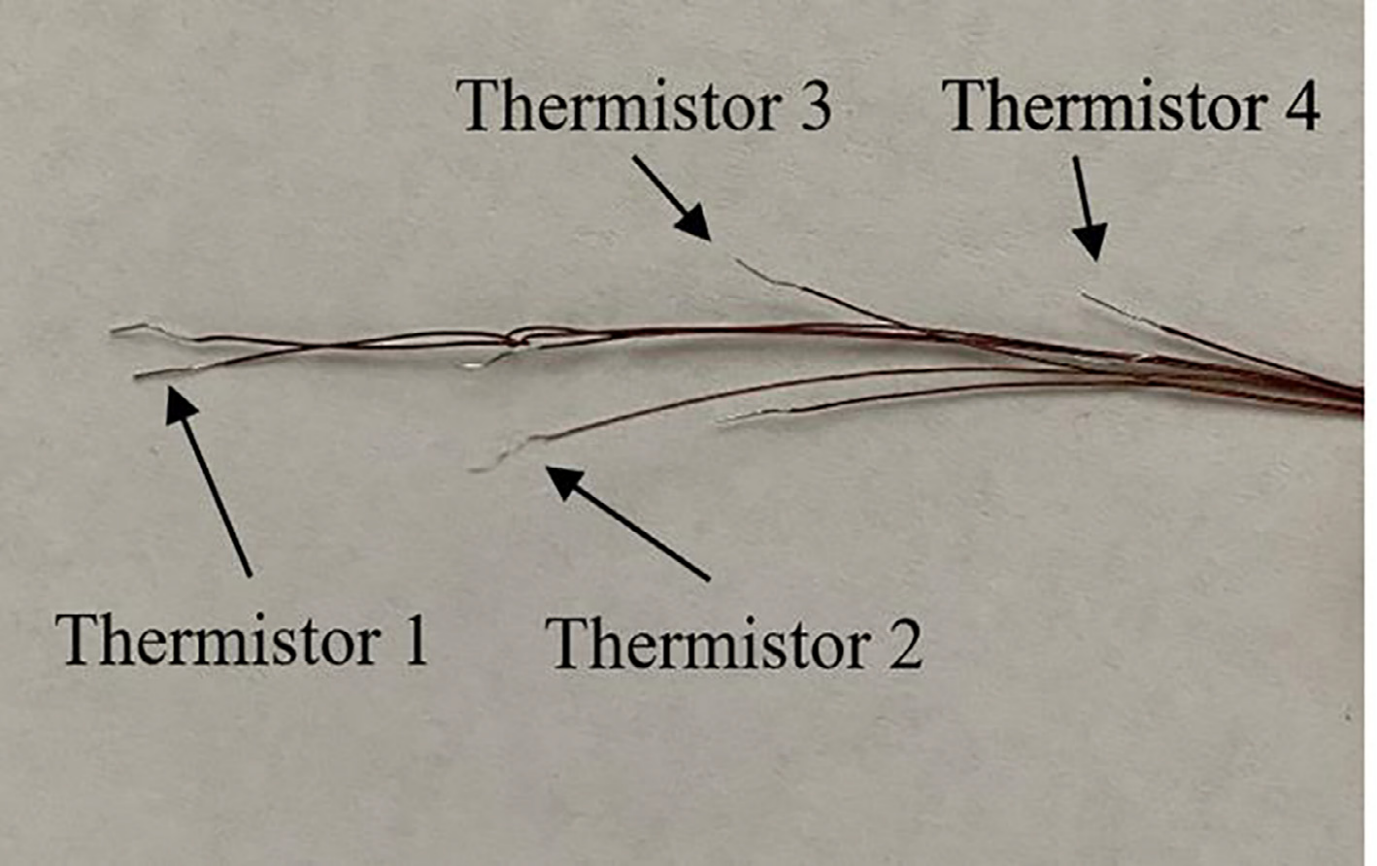
The leads of four thermistors after sealing the base of the dispensing needle with epoxy. Note that the depth order of the thermistors can still be determined after dispensing needle is epoxied (refer to PowerPoint template).

**Fig. 8:**
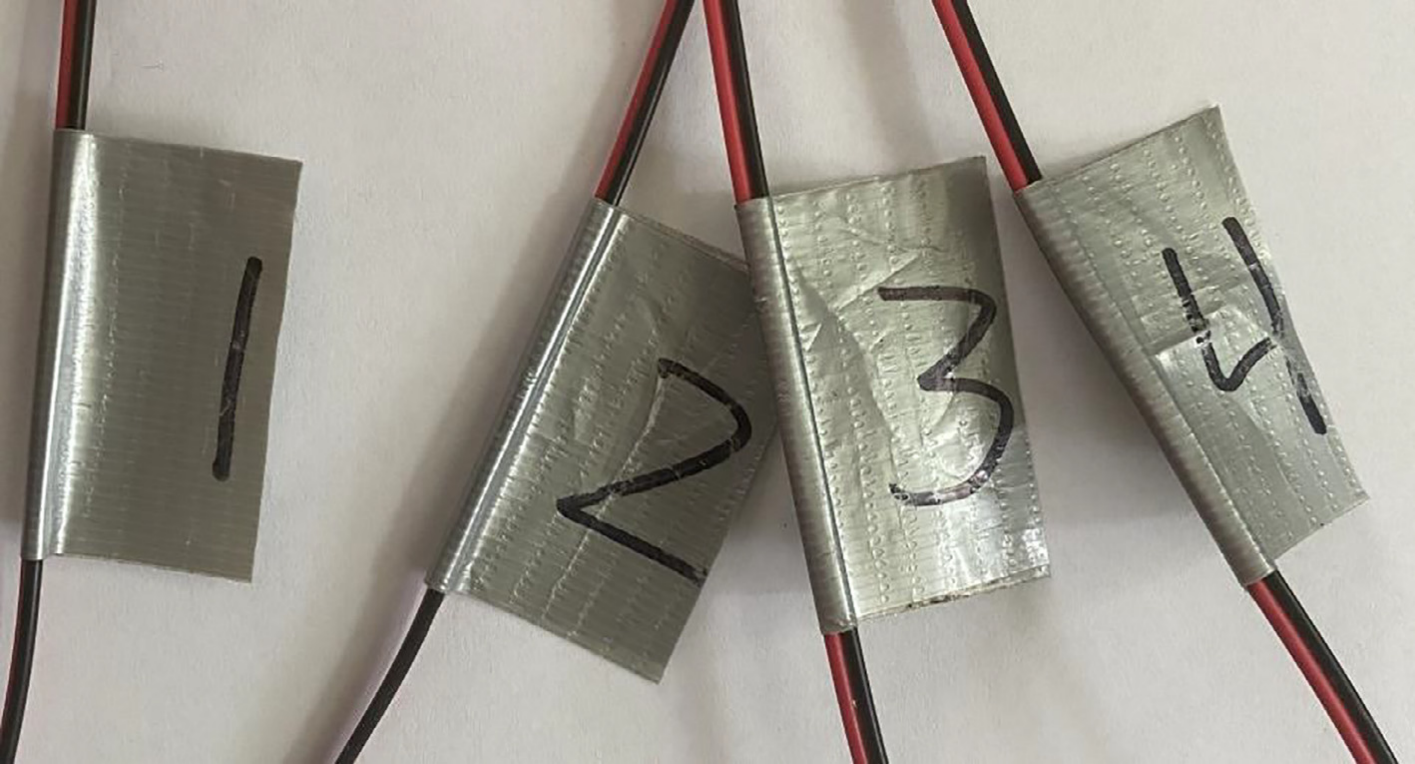
Labeled bonded extension wire. Each labeled extension wire corresponds with the thermistor number in Fig. 7.

**Fig. 9:**
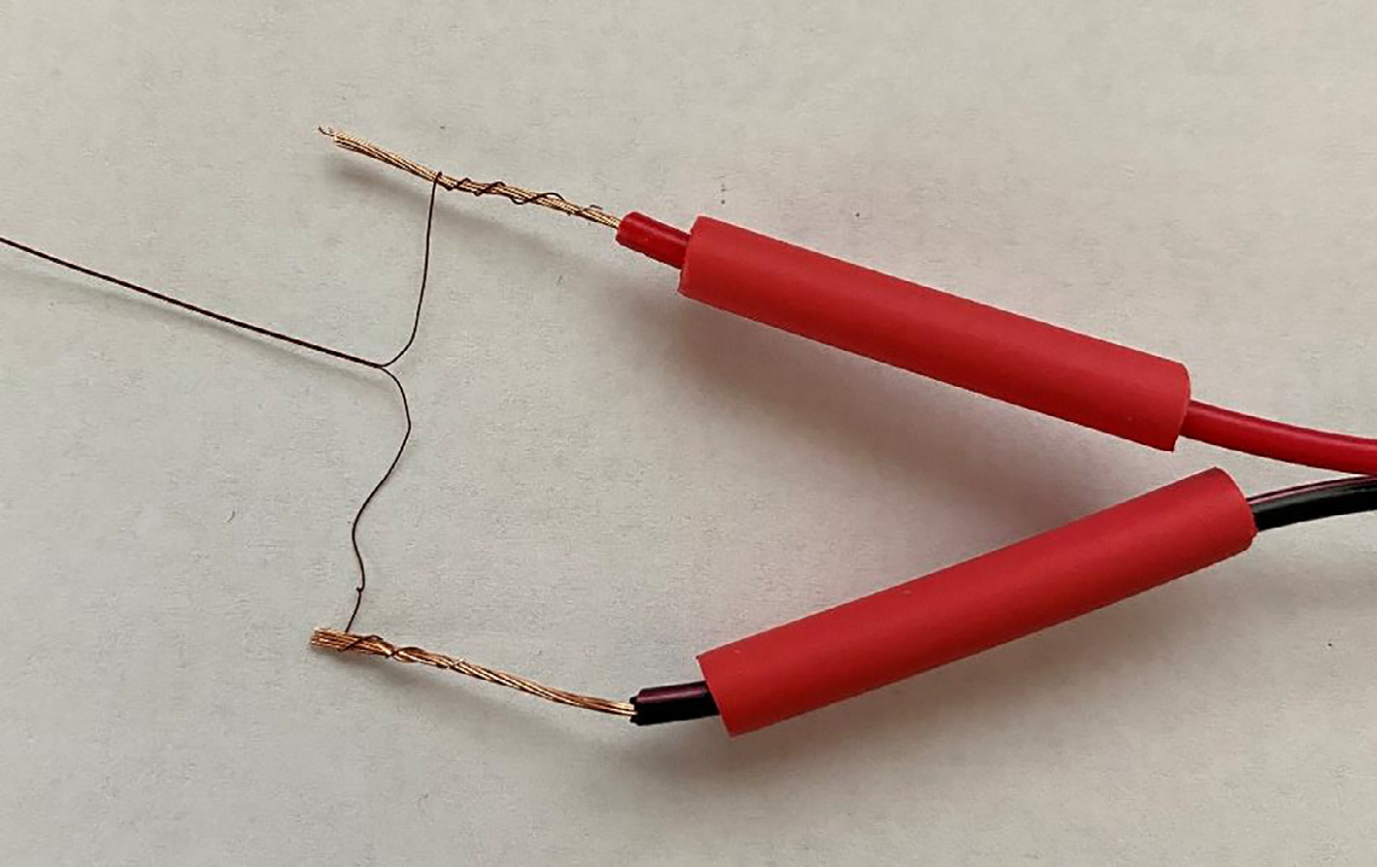
Connecting the thermistor leads to bonded extension wire. If using heat shrink wrap, be sure to put in place before soldering the thermistor leads to the bonded extension wire.
  g. *Secure Probe:* Using either the 3/8 in heat shrink wrap or electrical tape, sheath the probe from the base of the dispenser needle past the soldered connections to create a robust and secure probe for deployment (Fig. 10).

**Fig. 10:**
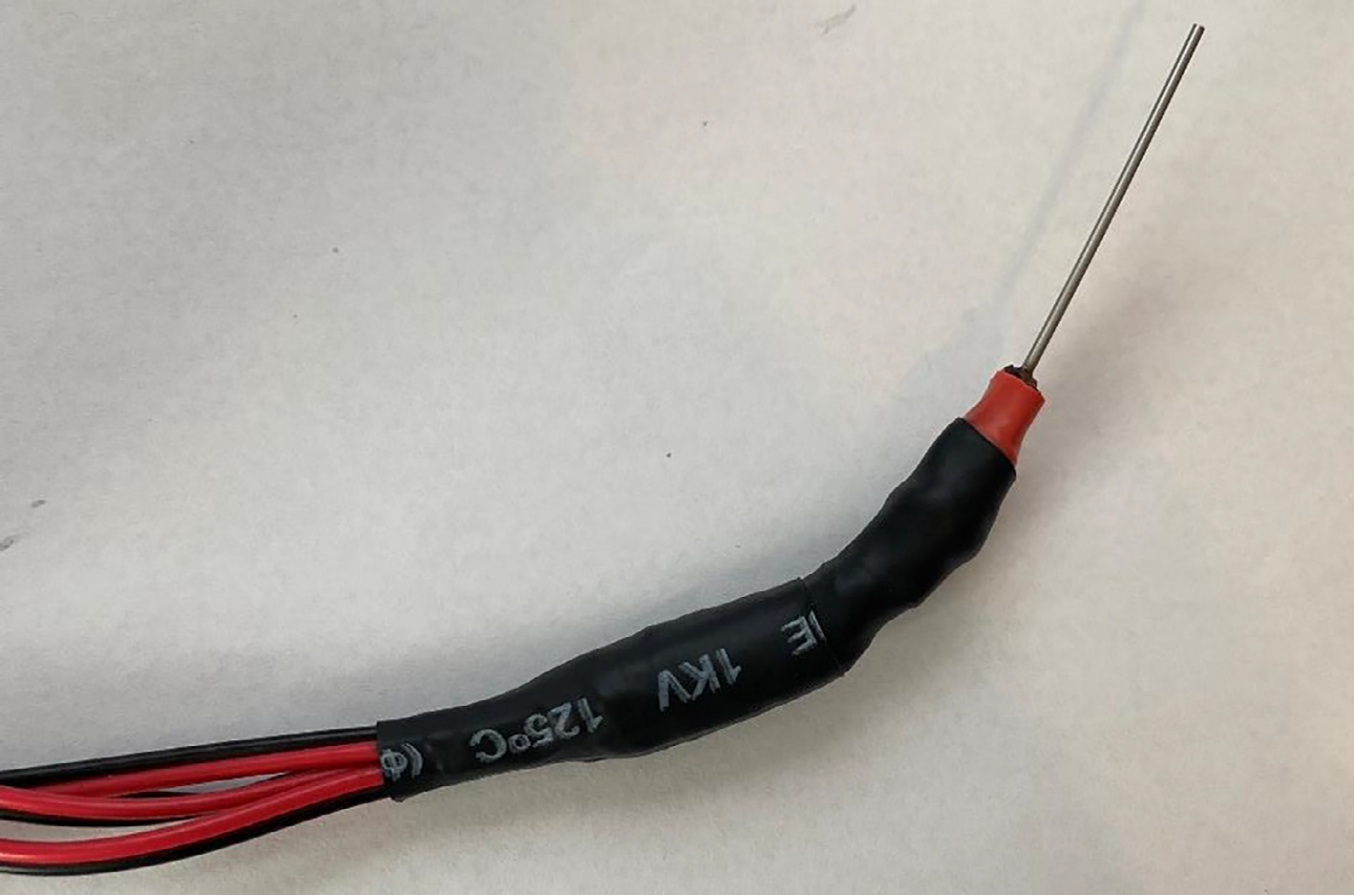
Completed reference probe with heat shrink wrap sheath. At this point, the only way to identify the thermistors is the labeling on the bonded extension wire.

**Fig. 11:**
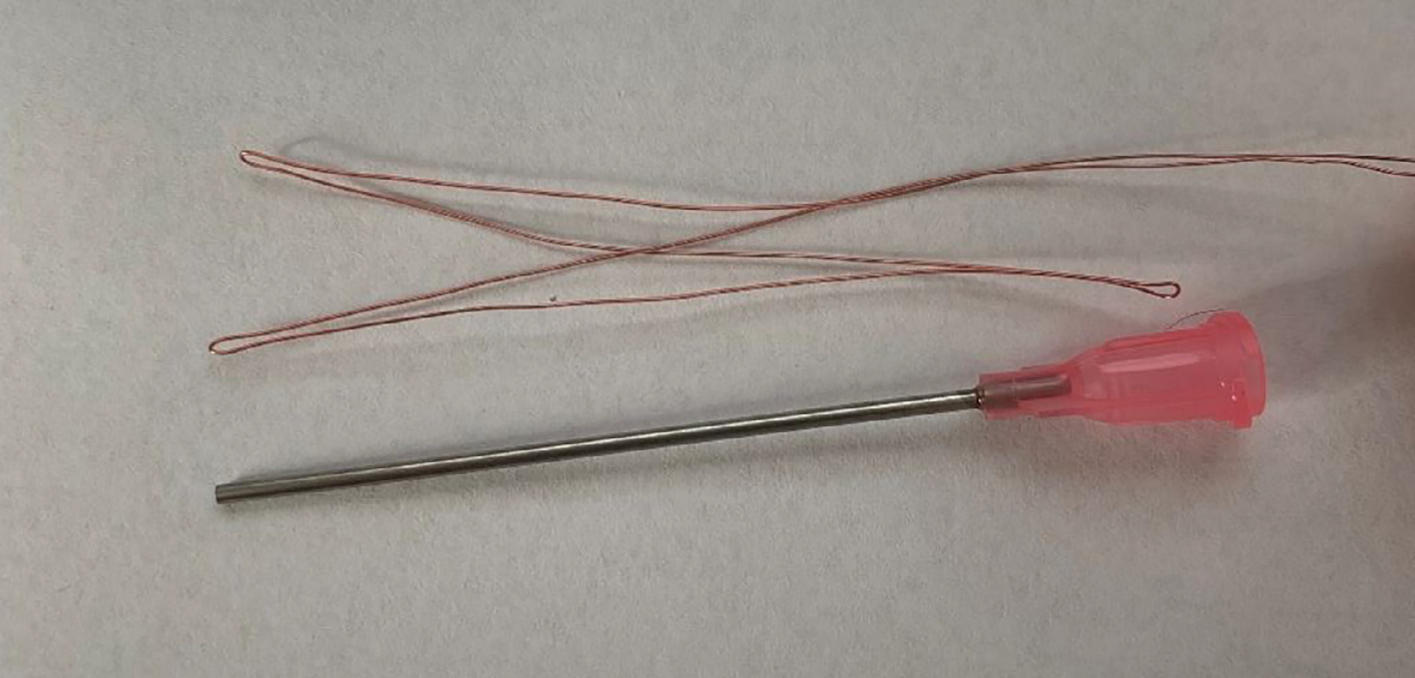
The “double loop” of heater wire prior to insertion into the dispensing needle.
  d. *Insert heater wire:* Beginning from the metal tip of the dispensing needle, insert the two ends of the heater wire segment and slowly push the heater wire through the needle. The fit should be tight, but manageable. Be sure to strip the two ends of the heater wire before soldering them to the bonded wire (Fig. 12). Excess heater wire should be removed to decrease the overall power demand of the heater probe.

2. Heater probes
  a. *Cut dispensing needle to desired length (optional):* The dispensing needles for the heater probes should be cut to match the length of the reference probes.
  b. *Cut heater wire to length:* Using the PowerPoint template, cut a ∼ 25 cm length of heater wire.
  c. *Double loop:* First fold the heater wire segment in half and then fold ∼ 5 cm back on itself, creating a “double loop” (Fig. 11).

**Fig. 12:**
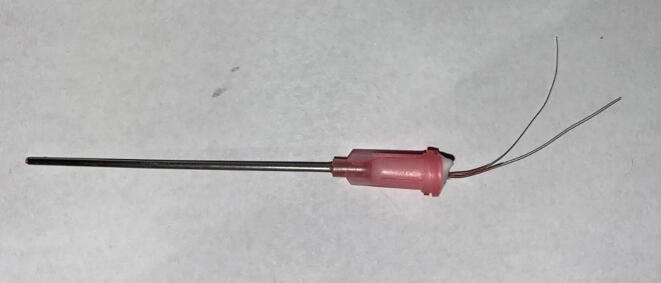
Epoxied heater probe. Note the stripped ends of the heater wire.
  e. Follow steps d-g from the previous section to complete the heater probes.

### Circuitry

**Fig. 13:**
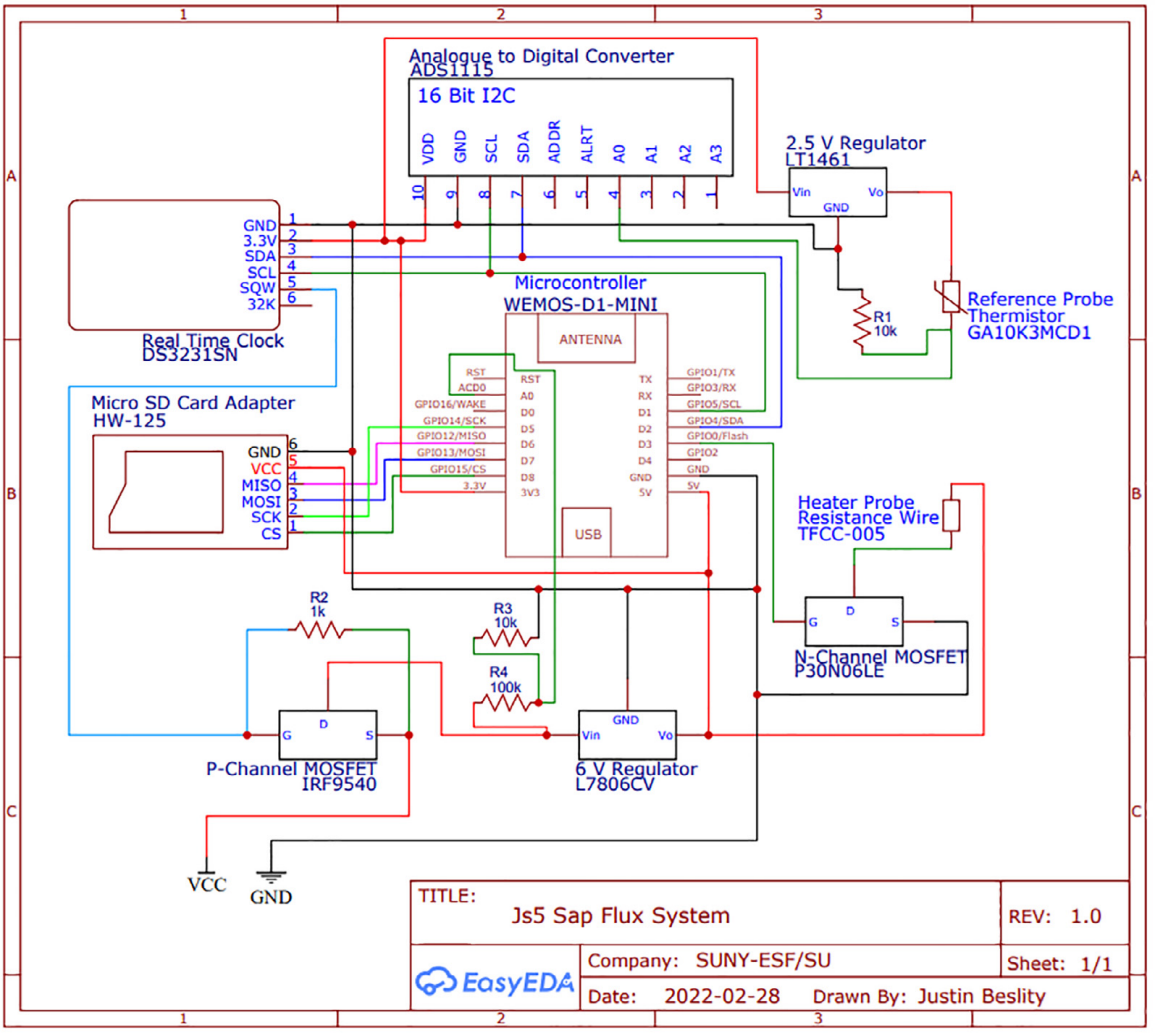
Simplified J_s_^5^ circuit schematic.

**Fig. 14:**
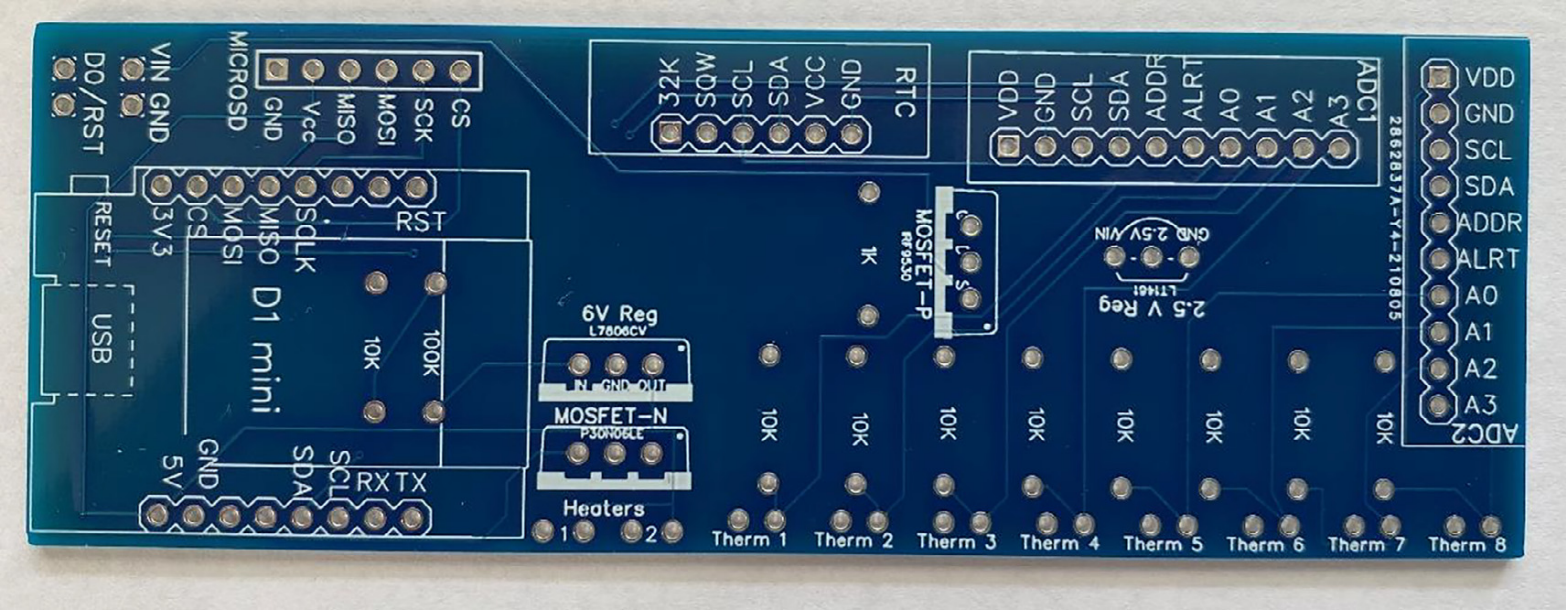
Printed circuit board for the J_s_^5^ showing the silk layer with annotated locations for each hardware component.

The circuitry (Fig. 13) is mounted on a fabricated PCB. The Gerber file listed in the design files table includes a silk layer marking the location of each hardware component (Fig. 14). Each board is designed to operate up to eight individual thermistors and two heater probes. In this example, we connect two four-thermistor probes and one heater probe to the printed circuit board. Most heat pulse velocity methods require both an upstream and downstream reference probe.

The recommended method to assemble the circuit boards is as follows:

1. *Resistors:* Place the one 1k, nine 10k, and one 100k Ohm resistors in their respective positions and solder in place (Fig. 15). Refer to the silk layer on the PCB when placing the resistors.

**Fig. 15:**
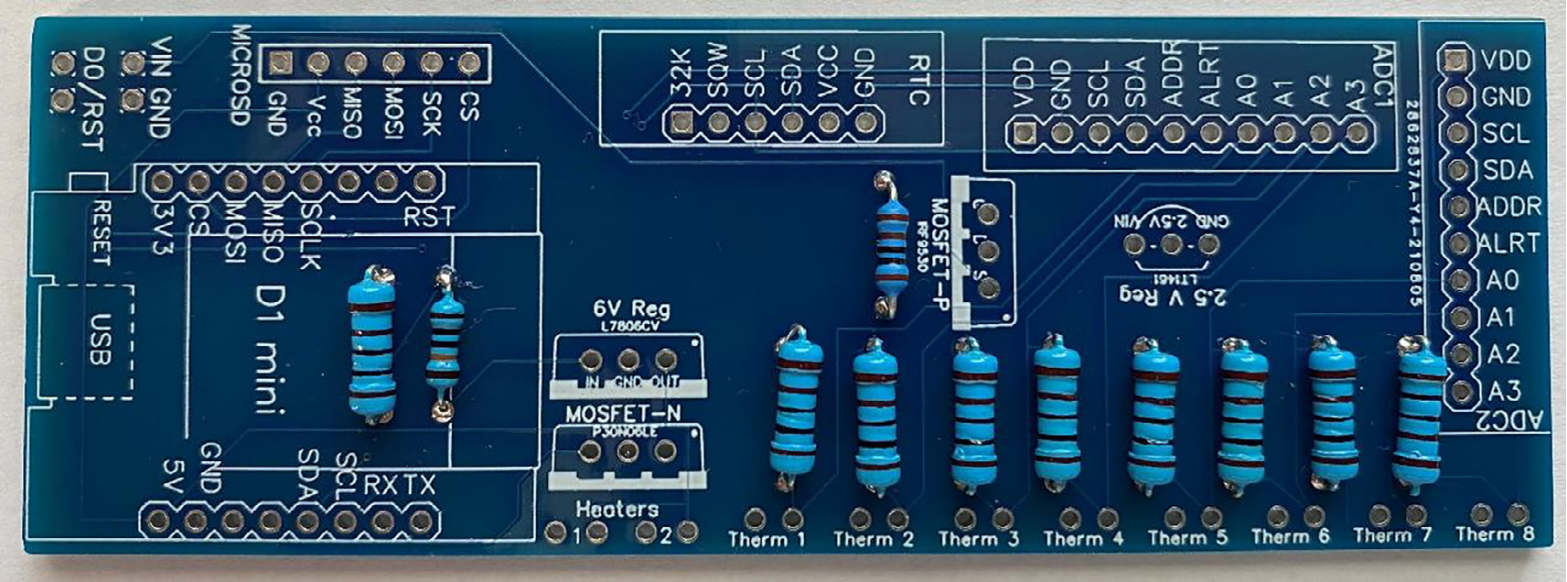
Printed circuit board with resistors in place.
2. *RTC modification:* The RTC DS3231SN requires slight modification to function as the alarm system for the J_s_^5^ [19]. Using the tip of the soldering iron, gently remove the resistors identified in Fig. 16.

**Fig. 16:**
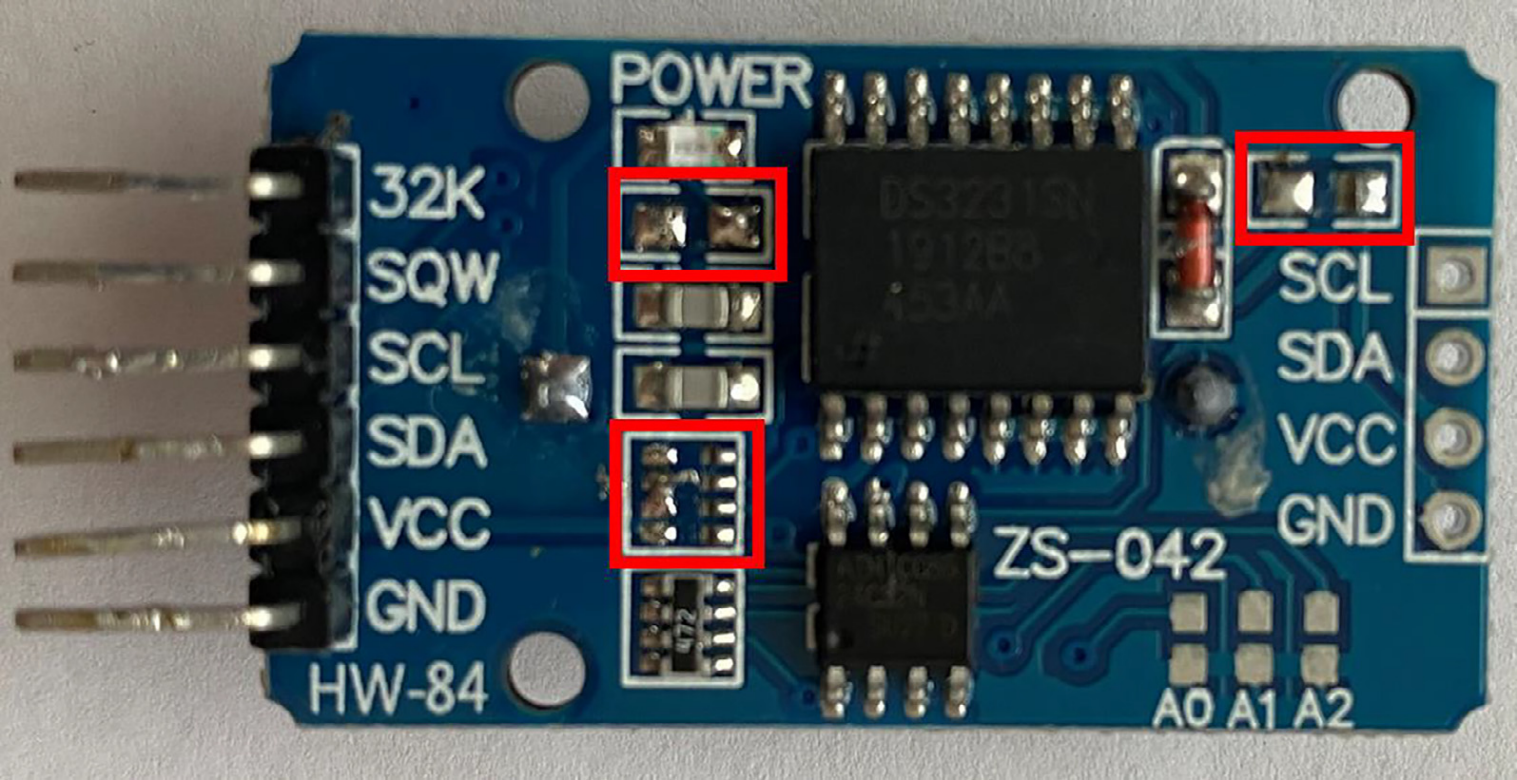
Modified RTC DS3231. Resistors (boxed in red) than need to be removed for the RTC to function as the alarm system for the J_s_^5^. (For interpretation of the references to color in this figure legend, the reader is referred to the web version of this article.)
3. *Components:* Place the two ADC1115 and solder in place, followed by the microcontroller (D1 mini), P and *N*-Channel MOSFETs, 6 V linear voltage regulator, 2.5 V voltage regulator, modified RTC DS3231SN, and finally the micro-SD card adapter module (Fig. 17).

**Fig. 17:**
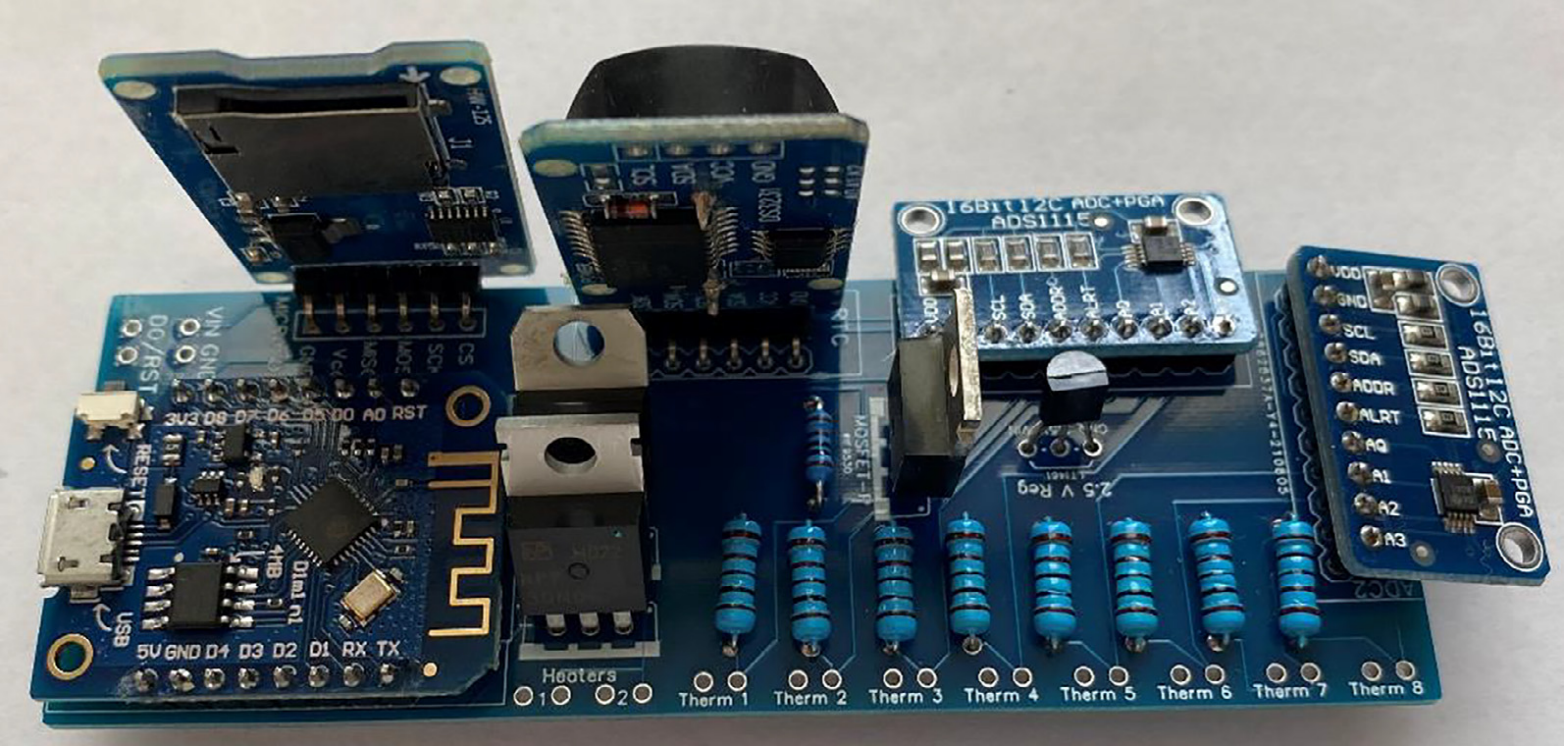
Printed circuit board with all hardware in place.
4. *Clean up:* Trim the leads of the resistors and other hardware components to create as flush a surface on the bottom of the PCB as possible. Be sure to avoid any potential electrical shorts (Fig. 18).

**Fig. 18:**
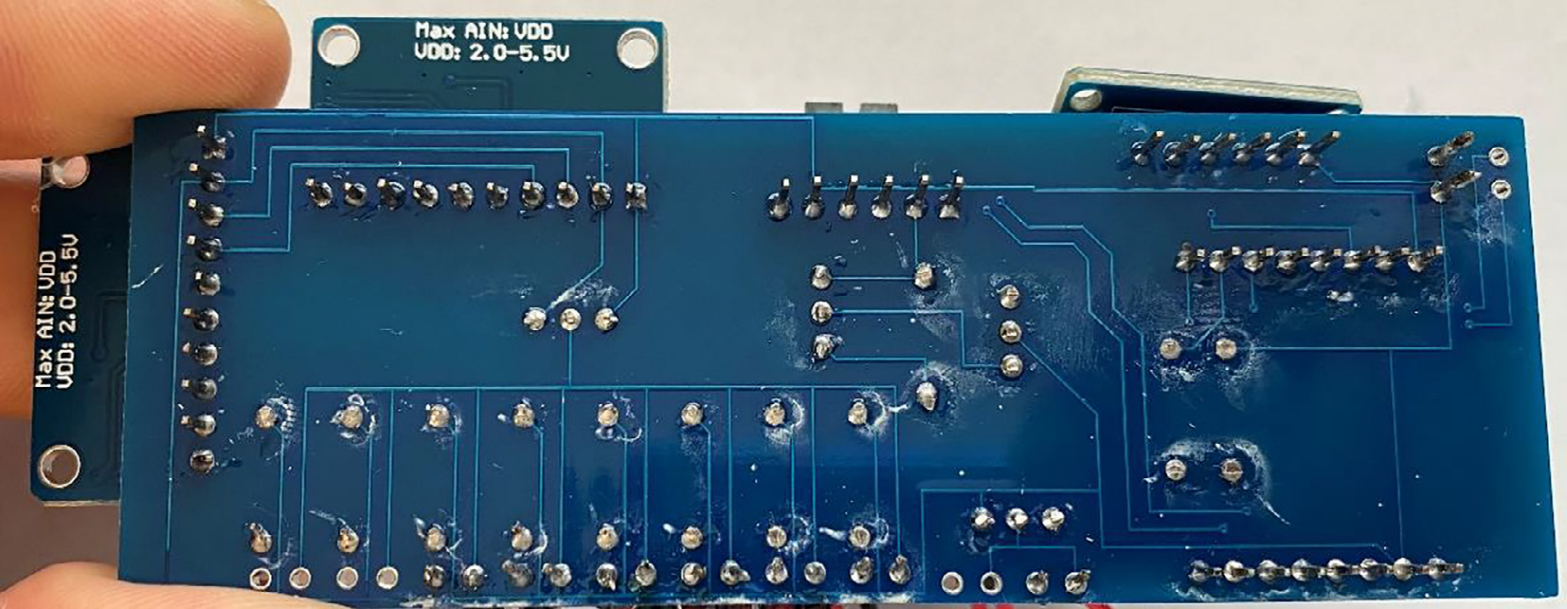
Printed circuit board with all hardware leads trimmed to prevent electrical shorts.

### Completing the device

**Fig. 19:**
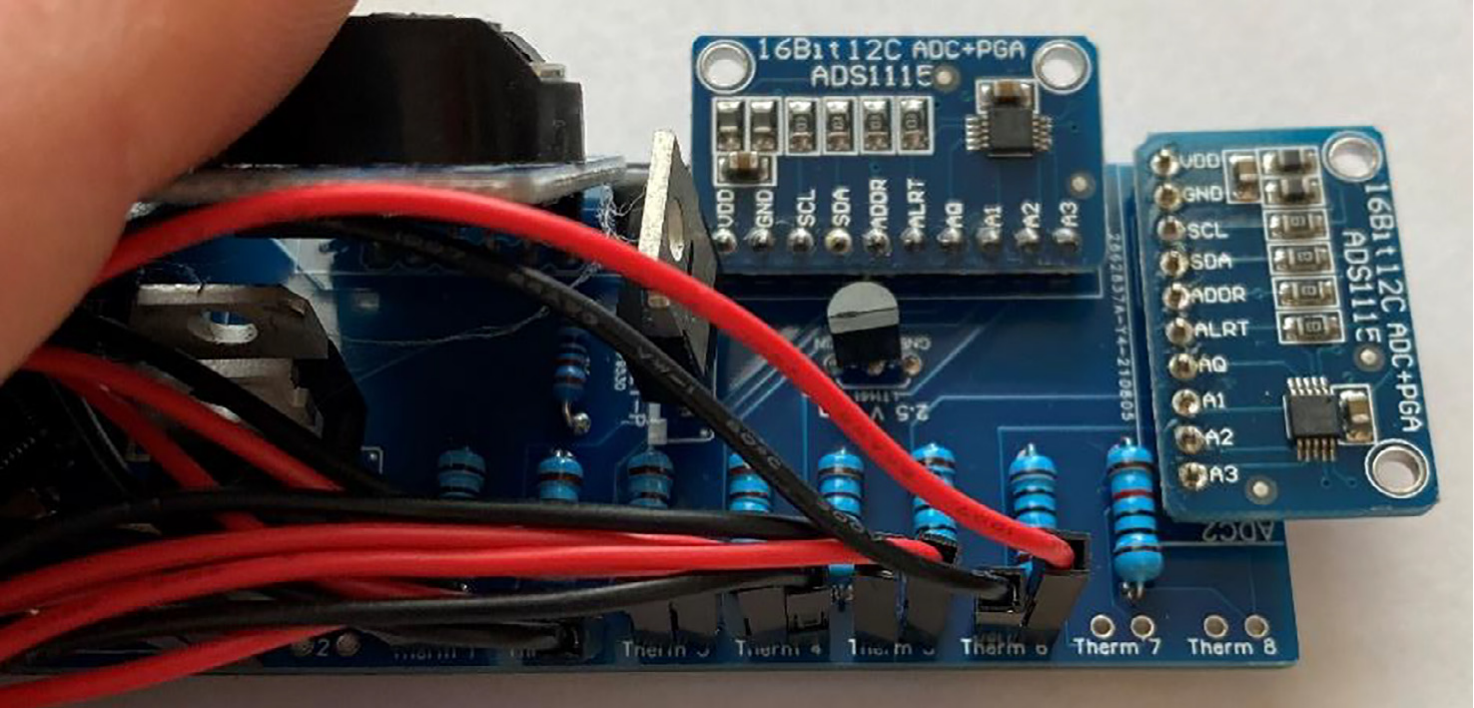
Connecting probes to the PCB.

1. *Attach probes to the PCB:* The thermistors in the probe attach to the circuit board so that thermistor 1 in the first probe is connected to the “Therm 1” pin holes, and thermistor 1 on the second probe is connected to “Therm 5” pin holes (thermistor is abbreviated as “Therm” on the printed circuit board; see Fig. 2). Strip away the insulation at the two ends of the extension wire opposite the dispensing needle. The exposed ends can be directly soldered to the printed circuit board, though we have found that the jumper wires listed in the bill of materials provided a robust connection to the circuit board which were able to withstand a full 7-month deployment in the field (Fig. 19). Compact connectors can be incorporated between the jumper wires and the extension wire to allow for easy replacement in the case of probe or circuitry malfunction (Fig. 20).

**Fig. 20:**
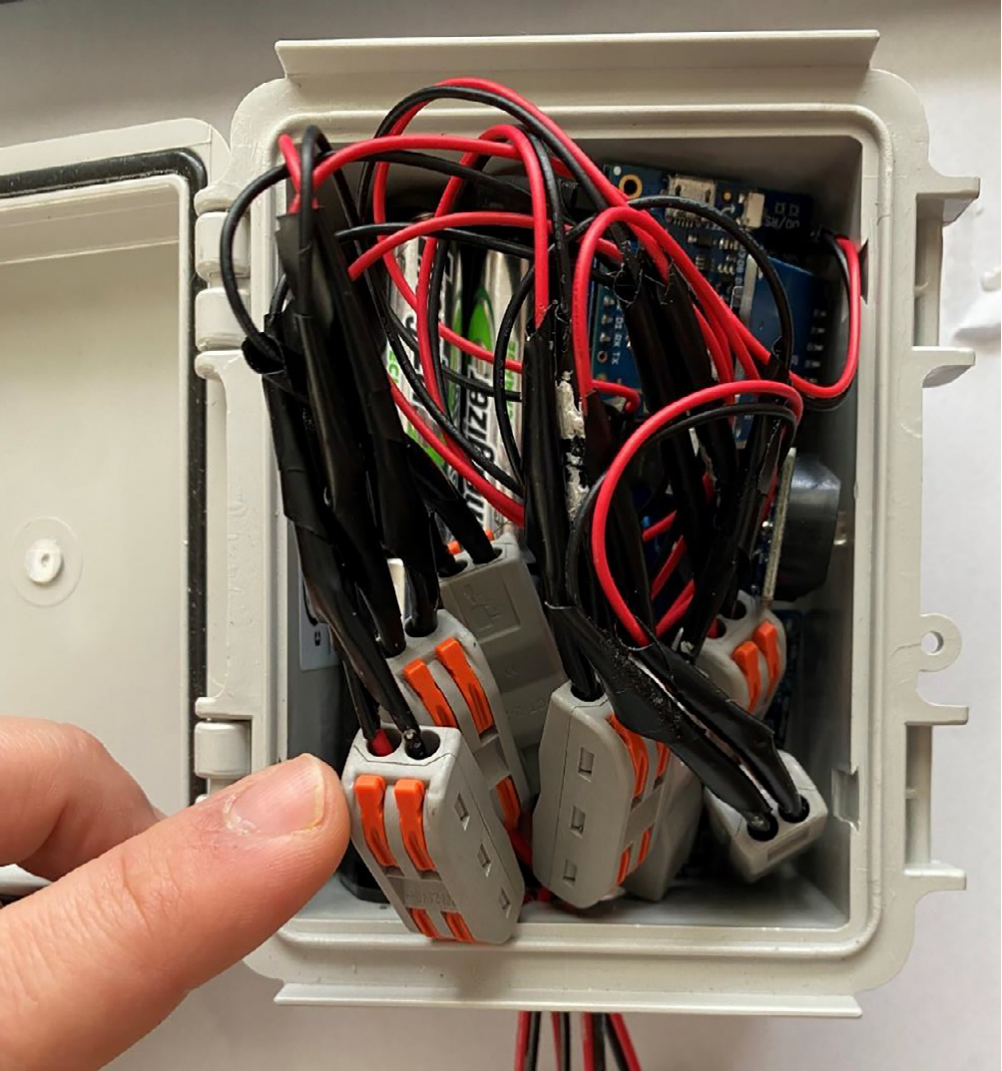
Compact connectors attached to each length of extension wire. If a probe or PCB malfunctions in the field, these connectors allow for easy replacement of the defective part.
2. *Batter Holder:* Solder black and red jumper cables to the corresponding leads of the 8x AA battery holder. Cover connections with heat shrink wrap. Solder the red and black jumper wires to the Vin and GND pins on the PCB, respectively (Fig. 21).

**Fig. 21:**
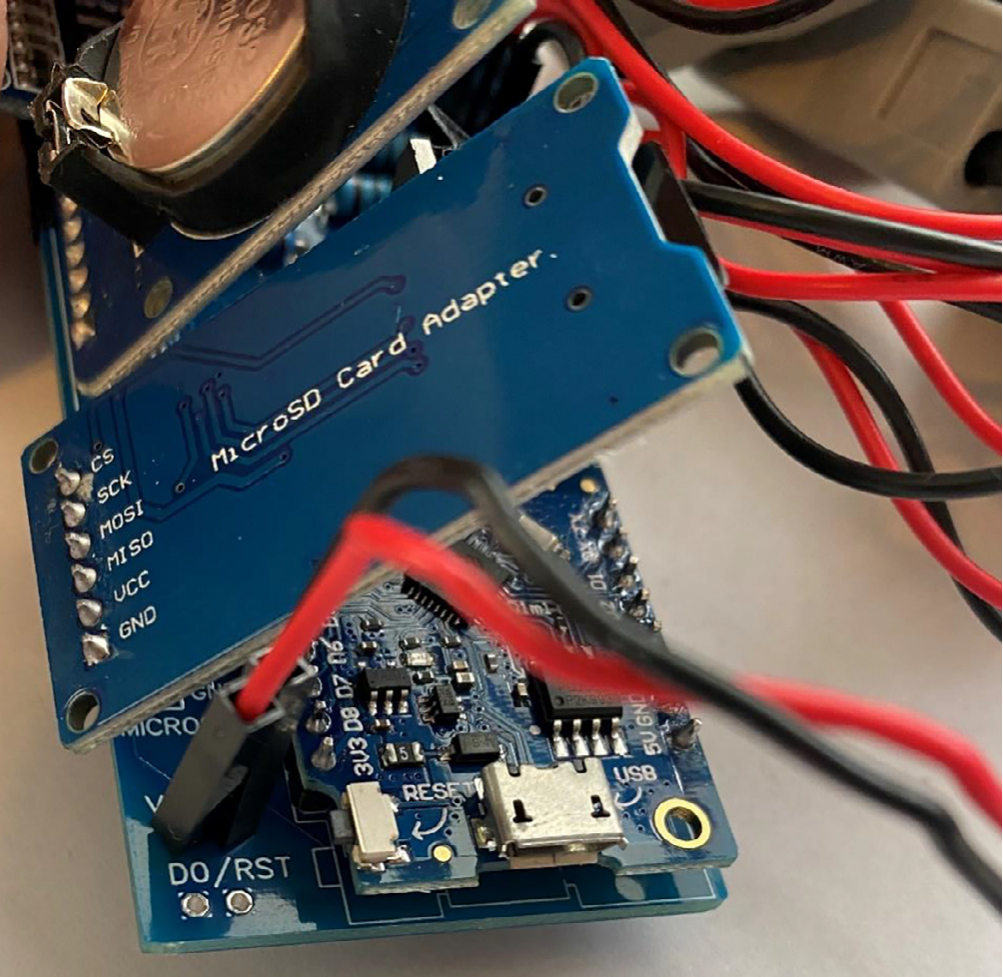
Connecting battery holder to the PCB.
3. *Drill an opening in the housing unit:* Using a 7/8 in hole saw drill bit, create an opening in the bottom of the Polycase housing unit (Fig. 22). Thread the probes through the hole and place the PCB and battery pack side by side within the housing unit.

**Fig. 22:**
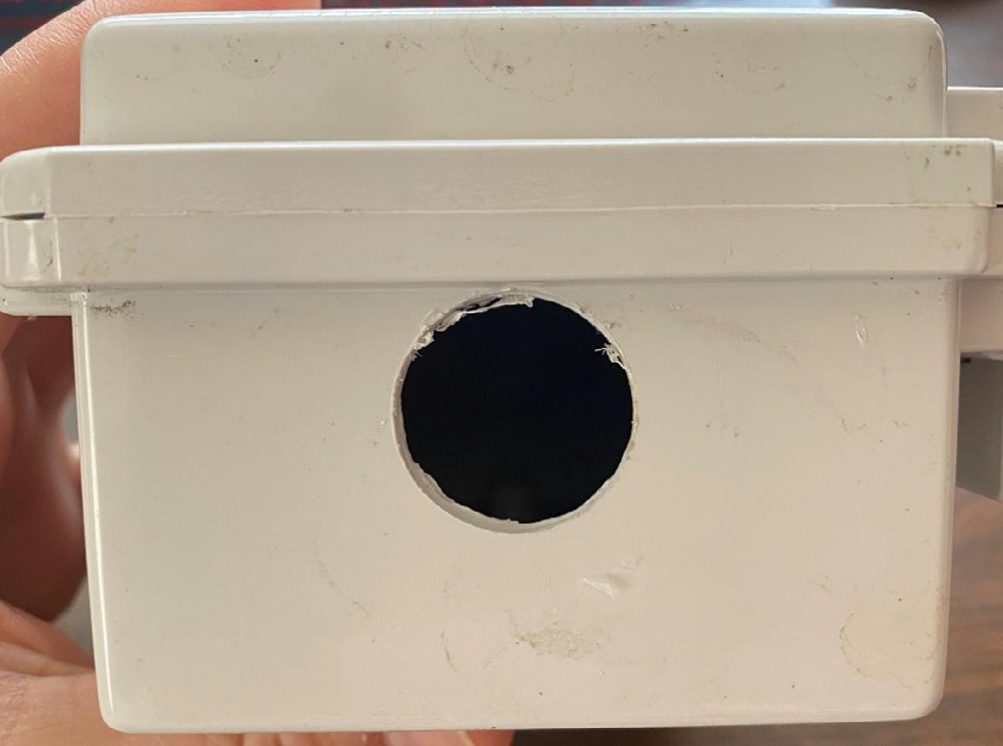
Housing unit (Polycase WH-02) with 7/8 in hole drilled in the bottom.
4. *Sealing the opening:* Seal the opening in the bottom of the housing unit with a small piece of poster tack, wrapping it around the extension wire and pressing it tight.

## Operation instructions

### Programing

The J_s_^5^ requires two programs to operate, the first to set the time on the RTC, as well as to format the files to be written to during operation, and a second which controls the measurement cycle.

1. Install Arduino IDE for your operating system (https://www.arduino.cc/en/software)
2. Open Arduino IDE
3. Go to File > Preferences
  a. In the Preferences pop up menu, copy the following into the “Additional Board Manager URLs” textbox.

https://arduino.esp8266.com/versions/2.3.0/package_esp8266com_index.json

4. Open Boards Manager from Tools > Board > Board Manager… menu
  a. Type “esp8266” into the text box.
  b. Select esp8266 by ESP8266 Community and click “Install”.
  c. Exit the Board Manager menu.
5. Open Tools > Board > ESP8266 Boards (2.7.4) and select your specific ESP8266 (e.g., Wemos D1 mini)
6. Open Tools > Manage Libraries… and download the following libraries by typing the names below into the textbox on the Manage Libraries… menu and clicking “Install”.
  a. Adafruit ADS1X15 by Adafruit
  b. RTClib by Adafruit
  c. SD by Arduino, SparkFun
  d. uRTCLib by Naguissa
7. Select the correct port in the Tools menu
8. Upload the Board_Configuration_SD program to the microcontroller using the Arduino IDE interface. Immediately upon upload, open the serial monitor to ensure the time is set properly and the SD card has initialized.
9. Upload the Write-Sap-Flow-Code_SD_Card program to the microcontroller. Once uploaded, and after the 5 s baseline temperature measurement, the LED on the microcontroller should light up for ∼ 2 s, indicating a heat pulse has initiated. Check the heater probe to see if heat has been generated.
10. Take out the micro-SD card and check the data to ensure all thermistors are operational.

The duration of the heat pulse, as well as the baseline and post heat pulse temperature measurements can be adjusted in the Write-Sap-Flow-Code_SD_Card.ino program.

### Installation

To install the J_s_^5^ in mature trees follow the protocol below:

1. Remove a small portion of the bark from the tree at breast.
2. Using a drilling template and the drill bit listed in the bill of materials, carefully drill the necessary holes in an area where the bark has been cleared. Number of holes, distance between holes, and depth of holes will depend on the method chosen. Improper drilling of holes is a common source of error [25].
3. Secure the housing unit to the tree within a close distance of the holes.
4. Apply the dielectric grease to the probes and carefully insert them into the tree.
5. Cover the probes with mylar shielding (see Fig. 1).

### Heat pulse methods

We tested our device, both in the laboratory and in the field, using the heat pulse (HP) method, including the Tmax and heat ratio variants of the heat pulse method.

Heat-pulse methods - based on the fundamental conduction–convection equation presented by Marshall (1958) - offer an alternative to the more commonly used thermal dissipation methods. Heat pulse methods measure the velocity (V_h_) of a short heat pulse (∼2 s) which can then be converted to SFD and whole-tree flow based on the physiological parameters of the sap wood. The approach assumes heat is conducted from a central heater probe down the tree to the “upstream” probe, and both conducted and convected up the tree to the “downstream” probe. Heat pulse velocities typically range from −20 to 200 cm hr^-1^ in the field, though values up to 500 cm hr^-1^ have been recorded in *Eucalyptus mannifera* [26].

There are several heat pulse methods, each with their strengths and drawbacks [27]. We used the Dual Method Approach (DMA) to gauge the devices’ capacities for measuring a wide range of flows [28]. This method combines the Heat Ratio Method (HRM), capable of measuring low/reverse flows, with the Tmax method, capable of measuring mid/high flow rates. The HRM utilizes two sensor probes installed at equal distances up- and downstream of the heater probe to determine the heat velocity with the equation:

(1) $$V_{h,HRM}=\frac{k}{x}\ln\left(\frac{{\Delta T}_{d}}{{\Delta T}_{u}}\right)$$

where *V_h,HRM_* is the heat velocity (cm s^−1^), *k* is thermal diffusivity (cm^2^ s^−1^), *x* is the distance between the sensor probes and the heater probe (cm), and *ΔT_d_* and *ΔT_u_* are the changes in temperature upstream and downstream following the heat pulse (K), usually recorded at between 60 and 100 s [29]. This method has been further modified to account for differences in the positioning of probes and heat pulse duration [30]:

(2) $$V_{h,HRM}=\frac{2k\left(ln\left(\frac{{\Delta T}_{d}}{{\Delta T}_{u}}\right)\right)}{x_{d}+x_{u}}+\frac{x_{d}-x_{u}}{2\left(t-\left(\frac{t_{o}}{2}\right)\right)}$$

where t_o_ is the heat pulse duration and *x_d_* and *x_u_* are the distances between the heater probe and the down- and upstream sensor probes, respectively (see Fig. 2).

The Tmax method utilizes one downstream sensor probe with the equation:

(3) $$V_{h,Tmax}=\frac{\sqrt{x_{d}^{2}-4kt_{m}}}{t_{m}}$$

where *V_h,Tmax_* is the heat velocity (cm s^−1^), x is the distance between the heater and downstream sensor probe, and *t_m_* is the elapsed time (s) for the downstream sensor to reach the maximum temperature of the heat pulse [31]. As with the HRM, this method has been modified to account for differences in the heat pulse duration [32]:

(4) $$V_{h,Tmax}=\sqrt{\frac{4k}{t_{o}}ln\left(1-\frac{t_{o}}{t_{m}}\right)+\frac{x_{d}^{2}}{t_{m}\left(t_{m}-t_{o}\right)}}$$

The heat pulse velocity selected for the DMA is determined by the Pérclet number (β) defined as:

(5) $$\beta =ln\left(\frac{{\Delta T}_{d,max}}{{\Delta T}_{u,max}}\right)$$

$$\mathrm{If}\;\beta \leq 1\;Then\;V_{h}=V_{h,HRM}$$

$$\mathrm{Else}\;V_{h}=V_{h,Tmax}$$

where *ΔT_d,_*_max_ and *ΔT_u,_*_max_ are the difference between the pre-heat pulse and maximum post-heat pulse temperatures in the down- and upstream probes, respectively [26].

Wound corrections for these methods are widely established, allowing for more accurate results [29], [33]:

(6) $$V_{c}=BV_{h}$$

where *B* is a correction coefficient that corresponds to a specific wound size. Pulse velocity is converted to sap flux density (g cm^−2^ s^−1^), using measured properties of the wood [34]:

(7) $$\mathrm{SFD}=\frac{\rho_{b}}{\rho_{s}}\left(m_{c}+\frac{c_{\mathrm{dw}}}{c_{s}}\right)V_{c}$$

where *ρ_b_* is the dry wood density (g cm^3^), *m_c_* is the moisture content (g_water_ g_dry wood_^-1^), *c_dw_* is the specific heat capacity of the dry sap wood material (J g^−1^ K^−1^), and *c_s_* and *ρ_s_* are the specific heat capacity of sap and the sap density, often assumed to be the specific heat capacity and density of water, respectively.

Single point SFD measurements are converted to whole tree sap flow (F, g s^−1^) by weighting each measurement from each temperature sensor by the corresponding sapwood area [24]:

(8) $$F=\sum_{j=1}^{n}\pi \left(r_{j}^{2}-r_{j-1}^{2}\right){\mathrm{SFD}}_{j}$$

where *j* is the number of temperature sensors in each probe and *r* is the radius associated with the j^th^ temperature sensor.

## Validation and characterization

### Laboratory set up

Excised branches 5 to 8 cm in diameter were harvested from *Acer saccharum* (diffuse-porous) and *Tsuga canadensis* (softwood) at Hammond Hill State Forest (Dryden, NY). Branches were enclosed in a plastic bag with wet paper towel in the field to minimize cavitation. Immediately before the experiments, the branches were cut again under water to approximately 30 to 60 cm in length using a handsaw. It is possible that the length of some vessels exceeded that of the branch segments, though this would not impact the relationship between SFD and gravimetric flow rate once the vessels were recharged via positive pressure. The ends of each segment were shaved with a razor blade to remove potential damage to the xylem caused by the handsaw.

Sap flow was artificially generated in the laboratory by applying positive pressure to the branches. A 38 L pressure tank was partially filled with tap water, allowed to equilibrate to room temperature, and then pressurized using a hand pump. Pressure was maintained at near constant levels during experiments; periodic hand pumping throughout the experiment maintained pressure as measured with a digital pressure gauge (DPGA-05, Dwyer Instruments, Inc., Michigan City, IN, USA). The branch was secured to the canister using a rubber coupling and hose clamps. When the system was pressurized, water emerged only at the cut end of the branch, and flow rate was measured gravimetrically based on the water collected in five-minute time intervals. The J_s_^5^ was tested at four flow rates associated with 1, 3, 5, and 9 psi (induced by changing positive pressure), as well as a no flow condition. Measurements at each flow rate were repeated four or five times at each flow rate, depending on the ability to maintain a near constant flow rate, to quantify precision.

The probes were inserted perpendicularly to the surface of each branch to a depth of 3 cm. Temperature sensors were positioned at 0.5, 1.5 and 2.5 cm along the upstream and downstream reference probes to provide a weighted average sap flow rate. Bark was removed from the area the probes were inserted to ensure as much of the probe as possible was in the sap wood region. The reference probes were installed 0.5 cm up- and downstream of the heater probe using a custom drill template. Dialectic grease was used to ease the insertion and to improve contact between the probe and sapwood. The branch was held in a vertical position using a ring stand to prevent any preferential flow patterns possible when positioned horizontally. Before beginning experiments, water was run through the branch for approximately 10 min to allow the branch temperature to equilibrate with the water temperature.

The lab experiment included: 1) sampling the baseline temperature measurements for 5 s, 2) applying a 2-second heat pulse (typically 6 V) [16], [35], and 3) monitoring the heat pulse for 100 s at 0.5 s intervals. The mean moisture content (m_c_) and sapwood density (ρ_b_) from stem cores for *A. saccharum* and *T. canadensis* collected in the field were used to calculate the predicted SFD from the heat pulse velocity (V_c_) for each species. The SFD (g cm^−2^ s^−1^) measurements were then converted to sap flow (g s^−1^). The correlation between the predicted and gravimetric sap flow was evaluated using linear regression statistics.

Following each branch experiment, Safranin O solution (0.1%) was added to the pressure canister to obtain a measurement of sap wood area. The dye took approximately 5–15 min to pass through the branch, depending on the species. A small cookie was then cut from the stem segment and shaved with a razor blade. The cookie was scanned, and the area and depth of red dye was determined using Image J software (Image J, NIH, USA, https://rsbweb.nih.gov/ij/). This procedure was repeated for three branches from both species.

### Power consumption

To assess possible reductions in power consumption, a subset of runs on excised branches was conducted using heat pulses applied at both 6 V and 12 V. When assessing the device at 12 V, the L7806CV linear voltage regulator was replaced with a L7812CV linear voltage regulator (a 12 V linear regulator), and the number of batteries used to power the device was increased accordingly. Flow rates were varied between 0 and 2500 g hr^-1^ with 25 to 30 measurements made across the range for each branch. Estimated sap flow was compared to gravimetric sap flow for both the 6 V and 12 V sets of measurements to evaluate change in accuracy as the voltage is decreased. Heat pulses were applied for 2 s at both voltages. In accordance with Ohm’s Law and Joule’s Law, halving voltage was expected to lead to one-quarter the wattage drawn.

**Fig. 23:**
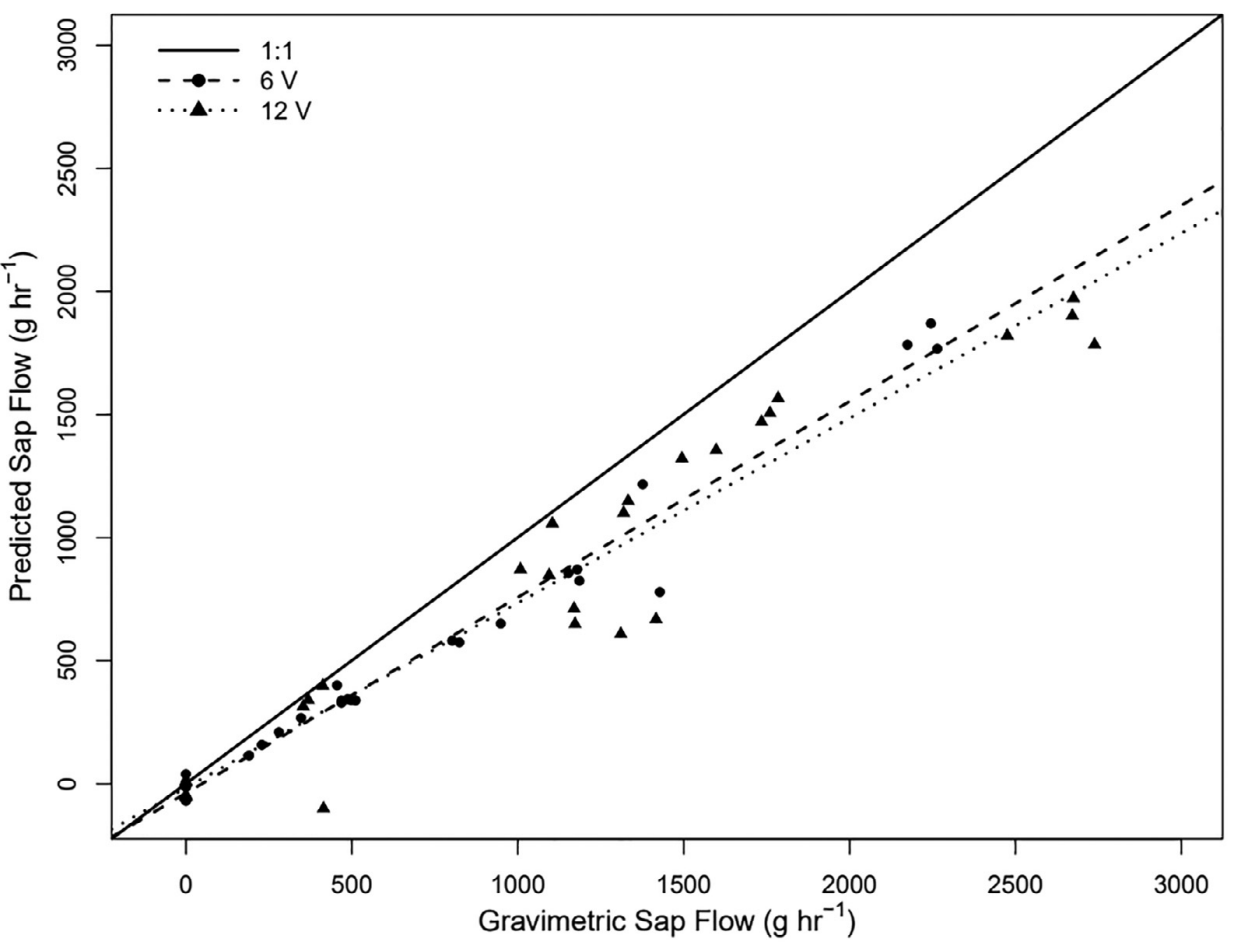
Correlation between the predicted sapflow measure at 12 V (triangles) and 6 V (circles) versus the gravimetric flow rate*.* The solid line represents a 1 to 1 relationship and the dashed and dotted lines represents the linear regression between the predicted and gravimetric sap flow for 6 and 12 V, respectively.

The sap flow predicted by both 6 V and 12 V pulses were comparable with no clear difference between them and a similar accuracy when evaluated against measured gravimetric flow (Fig. 23). The mean absolute errors of the predicted versus the measured values at 6 and 12 V were 201.1 (n = 25) and 278.8 (n = 30) g hr^-1^, respectively. The adjusted R^2^ for the correlation between the predicted and gravimetrically measured sap flow was 0.97 and 0.93 for 6 and 12 V, respectively. Given the similarity in predicted sap flow, 6 V pulses were presumed as suitable as 12 V pulses.

Device power usage was evaluated in order to quantify the period of time the system could be operated on a single battery charge (presuming 5 s of baseline temperature assessment, 2 s of heating, and 100 s of pulse monitoring). The Arduino based microcontroller drew 0.03 A during operation. Between measurements, the P-Channel MOSFET (IRF9540) gate is open, and the device draws no current. During the two second heat pulse, the heater wire drew 0.55 and 1.12 A at 6 and 12 V, respectively. By decreasing the applied voltage from 12 to 6 V, the corresponding power draw per heat pulse decreased from 13.44 to 3.30 W, resulting in a 24.6% increase in battery life. With eight 1.2 V 2000 mAh AA batteries, the system operated for 14 days if measurements are made every 30 min. The nominal power draw outside of the heat pulse was 0.2 W during the 105 s of temperature monitoring and 0 W outside of the measurement period. Actual field operation times varied between a low of 11 and a high of 21 days depending on the outside temperature, as well as the brand and rating of NiMH battery used. Longest operation times were obtained using 2300 mAh Energizer batteries.

### Laboratory assessment of sap flow measurement accuracy

**Fig. 24:**
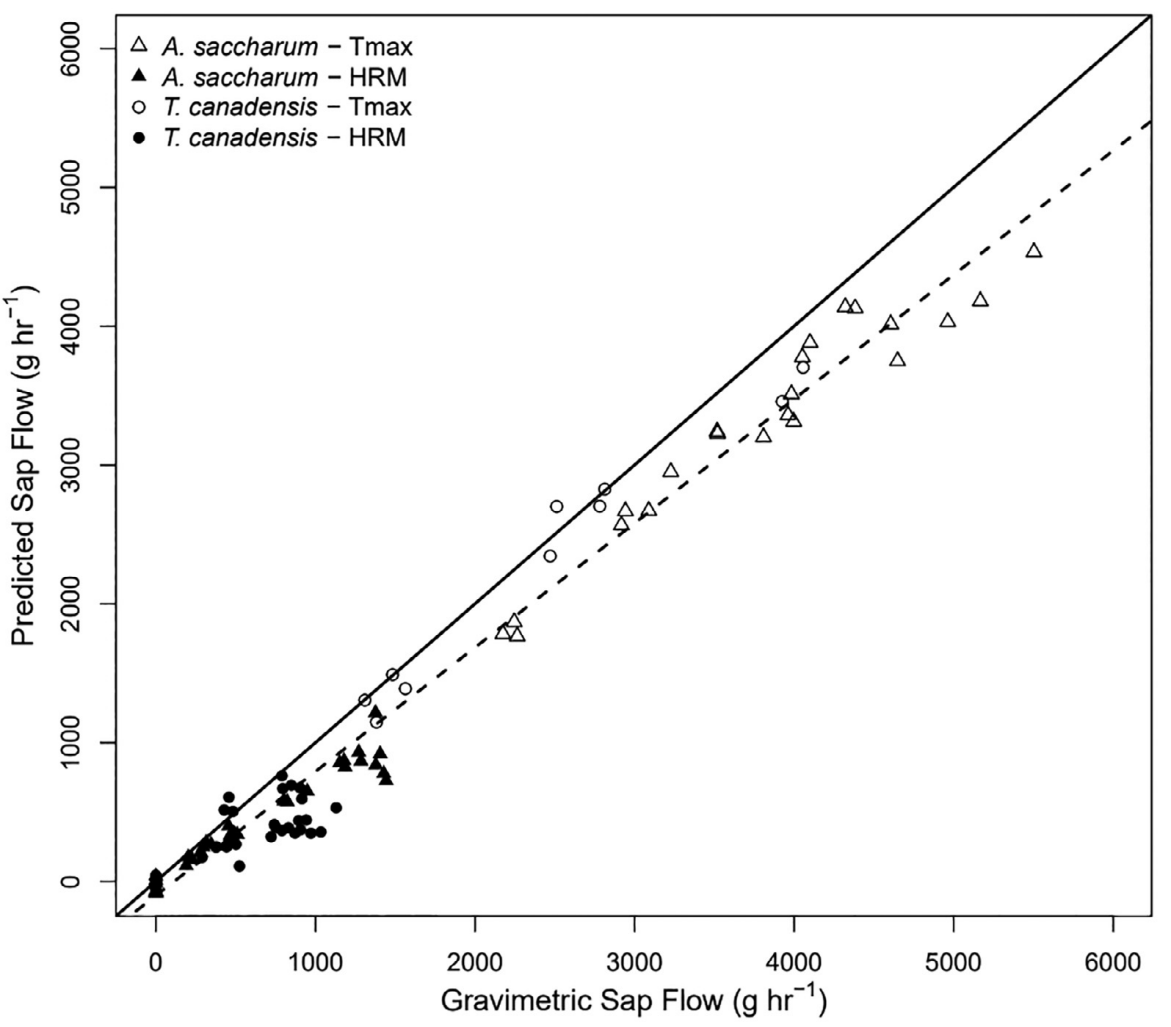
Correlation between the predicted sap flow measured via the HRM (solid) and Tmax (hollow) methods versus the gravimetric flow rate through branches of *A. saccharum* (triangle) and *T. canadensis* (circle)*.* The solid line represents a 1 to 1 relationship and the dashed line represents the linear regression between the predicted and gravimetric sap flow using three (0.5, 1.5 and 2.5 cm) radial measurement points.

With the DMA applied, sap flow estimated by the device closely matched that measured gravimetrically (Fig. 24). The relative error between device measurements and gravimetric measurements was 10.6% when aggregating across both species (Table 1) with a slight consistent underprediction in the device measurements. This underprediction is consistent with other studies and is often attributed to imperfect estimation of wounding effects [36]. Prediction accuracy was higher for *T. canadensis* than *A. saccharum* across all methods. The mean gravimetric flow rates for *T. canadensis* and *A. saccharum* were 829.0 and 1602 g hr^-1^, respectively. The lower flow rates in *T. canadensis* were reflected in the DMA algorithm; of the 63 independent measurements of sap flow in *T. canadensis*, 53 were identified as HRM (84%), compared to 25 of 57 in *A. saccharum* (44%). All ten Tmax measurements associated with *T. canadensis* occurred in one branch, which resulted in a lower mean absolute error and relative percent error than any other method in either species due to the repeated measurements at the same flow rate.

**Table 1:** Accuracy of each method in predicting gravimetric sap flow (g hr^-1^). Error is the percent deviation from the slope derived from the linear regression between each method and the associated gravimetric sap flow. MAE is the mean absolute error and the maximum and minumum sap flow ranges for each method are measured in sap flow (F; g hr^-1^).

**Method**	**Error (%)**	**adj R^2^**	**MAE (g hr^-1^)**	**Maximum F (g hr^-1^)**	**Minimum F (g hr^-1^)**
**Gravimetric**				5503	0
**HRM**	36.6	0.71	199.8	1217	0
*A. saccharum*	55.8	0.70	193.1	1217	0
*T. canadensis*	19.8	0.71	205.2	762.4	0
**Tmax**	15.1	0.93	437.2	4534	649.4
*A. saccharum*	13.4	0.93	491.9	4534	649.4
*T. canadensis*	9.7	0.96	164.6	3703	1149
**DMA**	10.6	0.96	292.6	4534	0
*A. saccharum*	12.8	0.96	345.7	4534	0
*T. canadensis*	6.0	0.95	197.5	3703	0

The motivation for using the DMA is clear when evaluating the suitability of HRM and Tmax methods applied separately. The HRM method had a maximum sap flow measurement of 1200 g hr^-1^ and was not able to resolve medium or high flow rates (Table 1). In contrast, the Tmax method was unable to resolve low flow conditions with the minimum flow measurement around 494 g hr^-1^. Besides limitations in range, the HR method had a 36.6% error and the Tmax method had a 15.1% error. While the percent error was higher for the HR method than the Tmax method, the overall magnitude of sap flow was lower which resulted in a lower overall MAE for the HR method. In a meta-analysis of every peer-reviewed published paper where the heat pulse velocity method was compared to independent measurements of plant water use, the mean R^2^ values for the HR, Tmax and Dual methods were 0.916, 0.859 and 0.892, respectively, which are comparable with or exceeded by the values of 0.71, 0.93, and 0.96, found in this study when applying the DMA [36].

### Field deployment

**Fig. 25:**
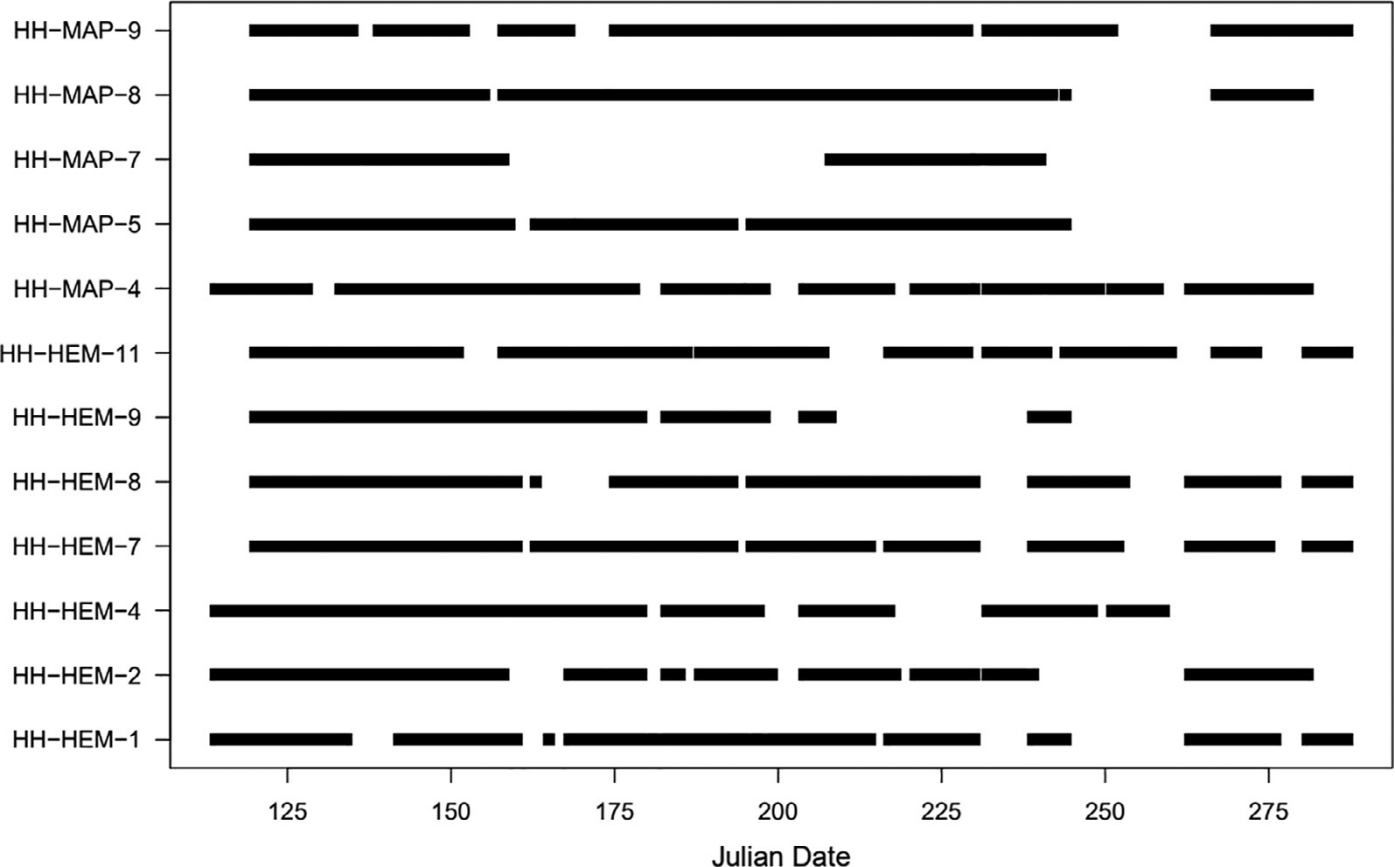
Time series demonstating the operations of 12 deployed custom sap flow sensors.

The operating periods of the 12 field-installed devices over the field season are shown in Fig. 25. On average, devices were operational 68% of the field season. Small data gaps generally occurred due to depletion of batteries and could be eliminated by more consistent replacement of batteries. Larger gaps typically reflect SD card malfunction, as seen in HH-MAP-9, HH-MAP-8, and HH-HEM-2. In some cases, data corruption was not identified for several weeks while the system otherwised appeared to be functioning properly, resulting in the very long gaps associated with HH-MAP-7 and HH-HEM-9. Additionally, if SD card malfunction was identifed after Sept. 15 (Julian Day 258), the device was removed for the rest of the year, as seen in HH-MAP-7, HH-HEM-4, and HH-HEM-9. In one case, HH-MAP-5, user error resulted in destruction of the device (i.e., battery pack connected to wrong wires), which was then decommisioned for the remainder of the deployment period.

Outside of user error, the issues resulting in data gaps have been resolved. The SD card malfuntion was identified as both a software and hardware issue; the software was adjusted to more effectively write to the SD cards and SD cards specifically designed to operate in outdoor environments were installed. More frequent field visits have eliminated the small gaps in data due to battery depletion.

For two trees, sap flow measurements from the field deployment were compared with vapor pressure deficit (VPD) measurements and seasonal phenological variations in order to assess whether sap flow measurements were physically consistent with expected controls. VPD is often presumed to be a dominant control on transpiration and correlation with VPD is indicative of reasonable operation of the sap flow probes.

**Fig. 26:**
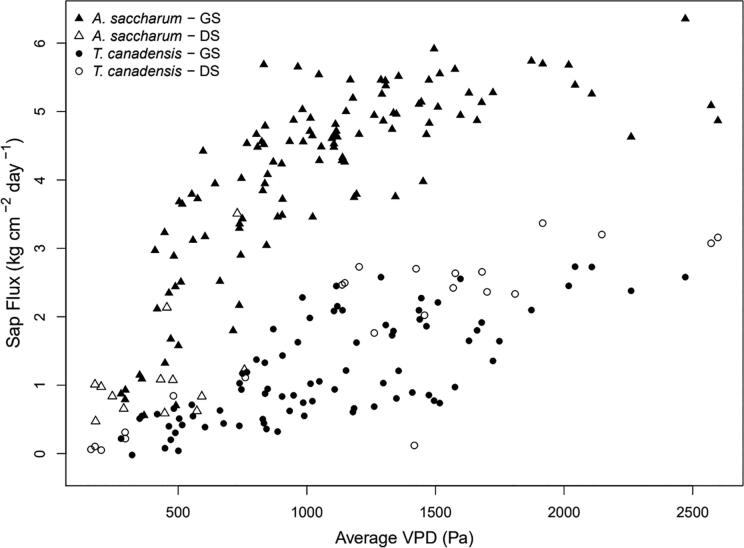
Correlation between daily sap flux and mean daily vapor pressure deficit (VPD) between 8:00am and 4:00 pm in in one individual of *A. saccharum* (triangle) and *T. canadensis* (circle) (HH-MAP-4 and HH-HEM-4, respectively; see Fig. 4). The data is classified by measurements collected during the growing season (GS; between 5/20/2021 and 10/3/2021 for *A. saccharum* and 6/1/2021 and 9/29/2021 for *T. candensis*; solid), and during the dormant season (DS; before 5/20/2021 and after 10/3/2021 for *A. saccharum* and before 6/1/2021 and after 9/29/2021 for *T. canadensis*; hollow).

The relation of VPD with estimated sap flow displayed seasonal changes consistent with phenological changes in the trees. Within the growing and dormant seasons, estimated sap flow strongly correlated to VPD (Fig. 26).

The largest sap flow values for *T. canadensis* occurred prior to June 1. Before June 1, the deciduous canopy was not fully developed and *T. canadensis* received maximum solar radiation exposure and transpired at a high rate. After June 1, sap flow shifted down and remained lower through the end of the season. In *A. saccharum* higher relative sap flow values occurred between May 20 and October 3 when the canopy was developed and leaves were most active. When a piecewise linear regression between daily VPD and daily estimated sap flow is calculated by species and season, regression relationships are strong with an adjusted R^2^ of 0.65 and 0.61, and 0.70 and 0.65, for *A. saccharum* and *T. canadensis* in the growing and dormant seasons, respectively.

**Fig. 27:**
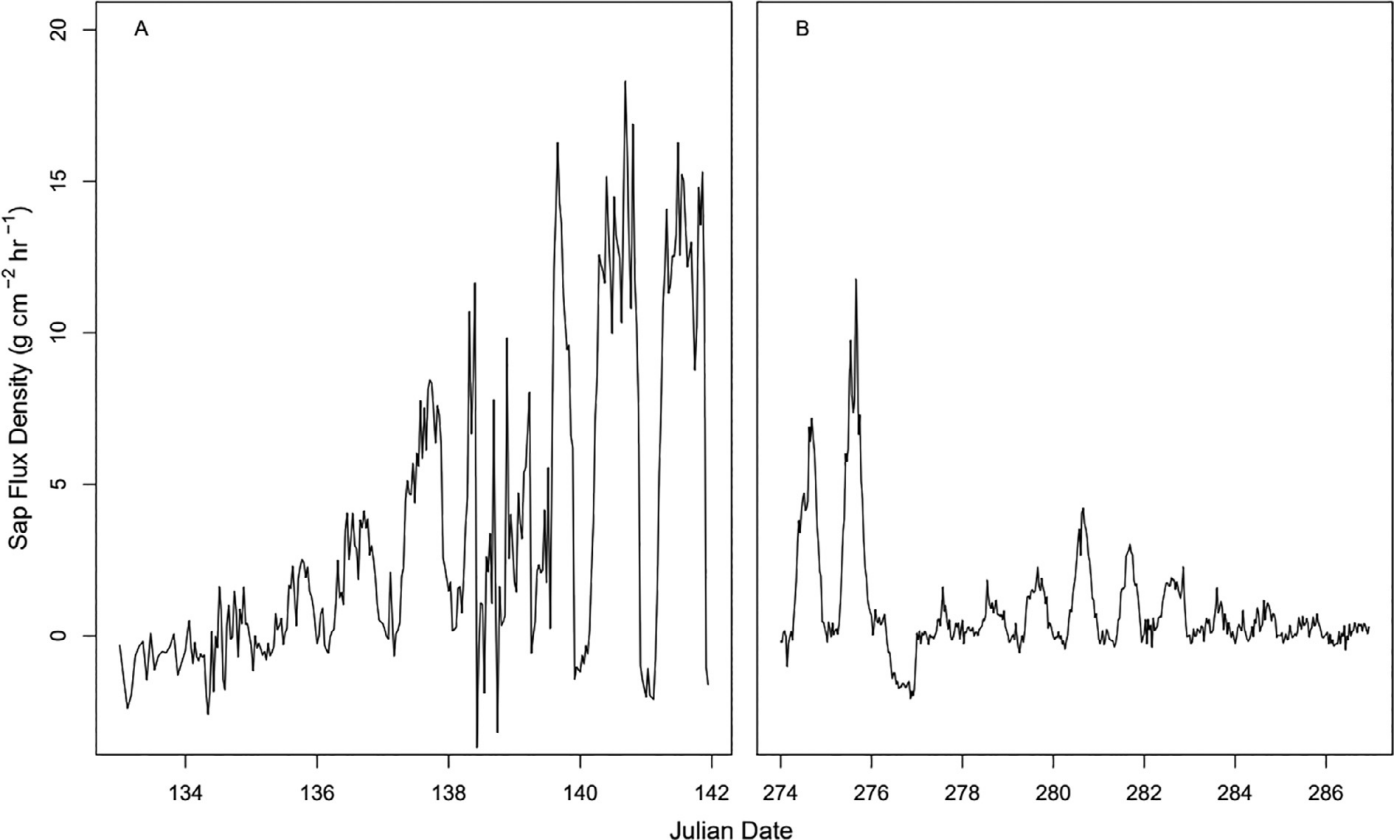
Time series of leaf out and senescense as measured by the J_s_^5^. Panels (A) and (B) depict the beginning and end of the growing season, respectively, in one individual of *A. saccharum* (HH-MAP-4; see Fig. 24).

To further show the sensitivity of the sap flow device to subdaily changes as well as seasonal changes, hourly sap flow for the beginning (Fig. 27.a) and end (Fig. 27.b) of the growing season are presented for the deciduous species *A. saccharum.* Time periods correspond to mid-May and early October. Sap flow increased progressively during the process of leaf out, and slowly declined as leaves changed color and senesced. Negative sap flux values indicate possible reverse flow scenarios. At the subdaily scale, there was an obvious diurnal variation in sap flow with values approaching or subceeding zero during nighttime.

### Potential flexibility for alternate field installations

By using rechargeable AA Ni-MH batteries, the J_s_^5^ can be self-contained within a small housing unit, allowing for simple and discrete deployment in the field. This device is ideal for urban site locations, where researchers may want equipment located higher up to avoid tampering, as well as remote site locations, where deploying and replacing large lead acid batteries may be prohibitive. In addition to the DMA detailed in this experiment, this device is capable of utilizing all HP methods, including the compensated heat pulse [33] and Sapflow+ [37] methods. Implementing the thermal dissipation method [38] is possible with modification to the probe design, however AA Ni-MH batteries are not suitable for this application.

The battery lifespan of the J_s_^5^ can be further increased through a variety of methods. Taking advantage of the functionality of the microcontroller, this device can be programmed to measure SFD at different time intervals (e.g., every hour or every half hour), skip certain hours (e.g., nighttime hours), or limit the length of the subsequent temperature measurements. The HP method requires only 5–10 s of background temperature measurements before the pulse is applied, and subsequent temperature measurements range from 100 to 400 s, depending on the HP method selected [29], [32], [33]. During field experiements, sampling every half hour, with the exception of 20:00–01:00 and 02:00–05:00, and measuring temperature for 100 s following the heat pulse, the J_s_^5^ was able to operate for 14 days before depleting the battery. This battery life could be increased further still by using a lead acid battery. Based on the device’s power consumption through Ni-MH battery tests, we could expect a lifespan of up to 100 days using a 12 V 18 Ahr lead acid battery under the standard sampling plan laid out in the methodologies. This would allow for multiple devices to be operated for long periods of time without the need to replace the batteries, as well as the inclusion of solar powered system for indefinite sampling.

## Conclusion

We developed a new device for measuring tree sap flow that provides a tenfold cost savings when compared to conventional commercial probes and dataloggers, as well as a 25% increase in battery life, without sacrificing accuracy. Including the cost of the rechargeable Ni-MH batteries, the J_s_^5^ costs < $150 when parts are bought in bulk. This offers an inexpensive, customizable, reliable, and easy to construct SFD measuring device that expands the types of possible experiments that could be implemented, including stand-scale transpiration estimates at the resolution of individual trees [39] and radial sap flux analyses [40]. When compared to a meta-data analysis of sap flow versus independent plant water use measurements, the J_s_^5^ performed comporably or exceeded the mean R^2^ values [36]. While the device functioned well, and predicted gravimetric sap flow accurately (adjusted R^2^ = 0.96, 10.6% error), improving the collection of physiological parameters, the alignment of probes during instrumentation, and quality of specific device components (such as SD cards) would lead to more accurate and consistent SFD measurements.

## Human and animal rights

No human or animal studies were conducted in this work.

## Funding sources

This project was supported in part by a CUSE grant from Syracuse University.

## Declaration of Competing Interest

The authors declare that they have no known competing financial interests or personal relationships that could have appeared to influence the work reported in this paper.
